# Butyrate potentiates *Enterococcus faecalis* lipoteichoic acid-induced inflammasome activation via histone deacetylase inhibition

**DOI:** 10.1038/s41420-023-01404-2

**Published:** 2023-03-28

**Authors:** Ok-Jin Park, Ye-Eun Ha, Ju-Ri Sim, Dongwook Lee, Eun-Hye Lee, Sun-Young Kim, Cheol-Heui Yun, Seung Hyun Han

**Affiliations:** 1 Department of Oral Microbiology and Immunology, and Dental Research Institute, School of Dentistry, Seoul National University, Seoul, 08826 Republic of Korea; 2 Department of Conservative Dentistry and Dental Research Institute, School of Dentistry, Seoul National University, Seoul, 03080 Republic of Korea; 3 Department of Agricultural Biotechnology and Research Institute of Agriculture and Life Sciences, Seoul National University, Seoul, 08826 Republic of Korea; 4 Institutes of Green Bio Science Technology, Seoul National University, Pyeongchang, 25354 Republic of Korea

**Keywords:** Mechanisms of disease, Bacterial infection

## Abstract

*Enterococcus faecalis*, a Gram-positive opportunistic pathogen having lipoteichoic acid (LTA) as a major virulence factor, is closely associated with refractory apical periodontitis. Short-chain fatty acids (SCFAs) are found in the apical lesion and may affect inflammatory responses induced by *E. faecalis*. In the current study, we investigated inflammasome activation by *E. faecalis* LTA (Ef.LTA) and SCFAs in THP-1 cells. Among SCFAs, butyrate in combination with Ef.LTA markedly enhanced caspase-1 activation and IL-1β secretion whereas these were not induced by Ef.LTA or butyrate alone. Notably, LTAs from *Streptococcus gordonii, Staphylococcus aureus*, and *Bacillus subtilis* also showed these effects. Activation of TLR2/GPCR, K^+^ efflux, and NF-κB were necessary for the IL-1β secretion induced by Ef.LTA/butyrate. The inflammasome complex comprising NLRP3, ASC, and caspase-1 was activated by Ef.LTA/butyrate. In addition, caspase-4 inhibitor diminished IL-1β cleavage and release, indicating that non-canonical activation of the inflammasome is also involved. Ef.LTA/butyrate induced Gasdermin D cleavage, but not the release of the pyroptosis marker, lactate dehydrogenase. This indicated that Ef.LTA/butyrate induces IL-1β production without cell death. Trichostatin A, a histone deacetylase (HDAC) inhibitor, enhanced Ef.LTA/butyrate-induced IL-1β production, indicating that HDAC is engaged in the inflammasome activation. Furthermore, Ef.LTA and butyrate synergistically induced the pulp necrosis that accompanies IL-1β expression in the rat apical periodontitis model. Taken all these results together, Ef.LTA in the presence of butyrate is suggested to facilitate both canonical- and non-canonical inflammasome activation in macrophages via HDAC inhibition. This potentially contributes to dental inflammatory diseases such as apical periodontitis, particularly associated with Gram-positive bacterial infection.

## Introduction

Apical periodontitis is an inflammatory disease around the apex of a tooth root [1]. The infiltration of bacteria into root canals is critical in the initiation of apical periodontitis [2]. *Enterococcus faecalis*, a Gram-positive bacterium, has been commonly found in periapical lesions [3]. Lipoteichoic acid (LTA) is an important etiologic agent of Gram-positive bacteria, which is considered as the counterpart of lipopolysaccharide (LPS) of Gram-negative bacteria [4]. LTA of pathogens can efficiently induce inflammatory responses by induction of pro-inflammatory cytokines and chemokines in the host [5, 6].

Commensal bacteria produce short-chain fatty acids (SCFAs) including acetate, propionate, and butyrate by the fermentation of dietary fibers [7]. SCFAs serve as a modulator of mucosal immune responses and the epithelial barrier function [8]. SCFAs can reduce intestinal diseases such as colitis and inflammatory bowel disease [9] and LPS-induced pro-inflammatory factors such as TNF-α and nitric oxide in rat neutrophils [10]. In contrast, SCFAs can facilitate inflammatory responses as butyrate increases the IL-1β in LPS-stimulated THP-1 cells [11]. SCFAs increase IL-6 and CXCL8 in TNF-α-induced human lung fibroblasts [12]. The concentration of total SCFAs in subgingival plaque samples is positively correlated with the degree of gingival inflammation [13]. Butyrate and propionate are frequently detected in the root canals of teeth suffering from apical periodontitis [14]. Therefore, LTA and SCFAs, both of which are commonly found in the apical lesions, may possibly cooperate to induce inflammation and subsequent apical periodontitis.

Inflammasomes are cytosolic protein complexes that mediate the innate immunity by releasing IL-1β and IL-18 [15]. Inflammasome activation is induced by two pathways: the canonical pathway mediated by caspase-1 and the non-canonical pathway mediated by caspase-4, caspase-5 (in human), and caspase-11 (in mice) [16]. Pathogen-derived ligands or endogenous danger signals can induce pro-IL-1β expression and NLRP3 activation. Subsequently, the activated NLR family pyrin domain containing 3 (NLRP3) recruits apoptosis-associated speck-like protein containing a caspase recruitment domain (ASC) and pro-caspase-1, which triggers assembly of the inflammasome complex. This leads to caspase-1 cleavage to the active form responsible for mature IL-1β secretion [17]. IL-1β induces periodontal tissue and bone destruction [18, 19]. IL-1β expression in gingival crevicular fluid is related to the severity of periodontal diseases [20]. The inflammasome activation and IL-1β expression are increased in periapical lesions with apical periodontitis [21, 22]. Thus, inflammasome activation induced by secretory microbial molecules may be important in the pathogenesis of apical periodontitis. In the present study, we investigated if *E. faecalis* LTA (Ef.LTA) in combination with SCFAs can induce the inflammasome activation and IL-1β production in THP-1, a human monocytic cell line commonly utilized in inflammasome research [23]. At this end, we confirmed the in vitro results in vivo using a rat apical periodontitis model [24].

## Results

### Ef.LTA in the presence of SCFAs substantially induces inflammasome activation

**Fig. 1:**
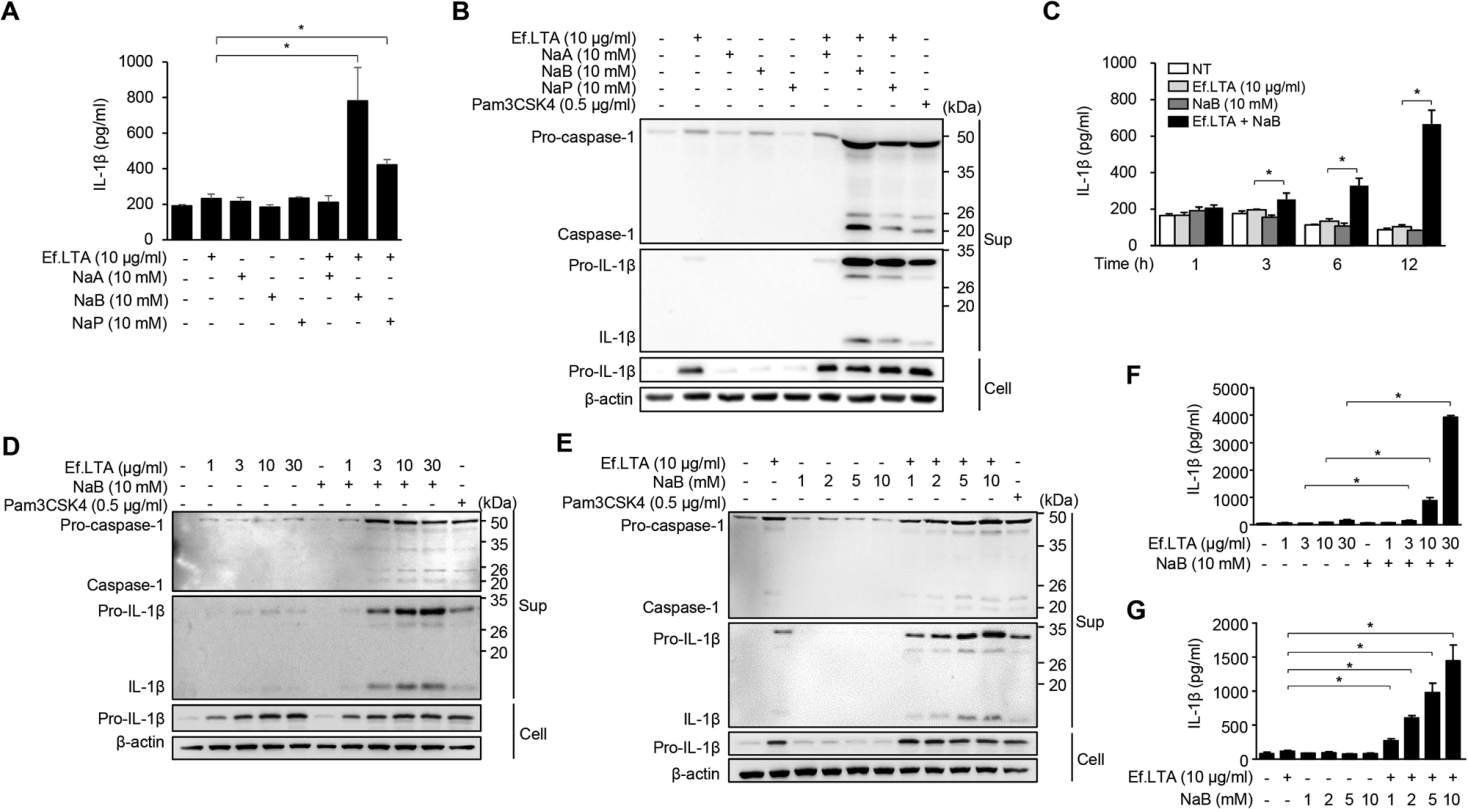
*E. faecalis* LTA together with butyrate potently induces IL-1β secretion and caspase-1 activation in human macrophages. **A**, **B** PMA-differentiated THP-1 cells were stimulated with 10 μg/ml of *E. faecalis* LTA (Ef.LTA) in the presence or absence of sodium acetate (NaA), sodium butyrate (NaB) or sodium propionate (NaP) for 6 h. **A** IL-1β expression in culture supernatants was determined by ELISA. **B** The pro- or active forms of caspase-1 and IL-1β in the culture supernatants (Sup) and pro-IL-1β and β-actin in the cell lysates (Cell) were detected by immunoblotting. **C** The cells were stimulated with indicated stimuli for various time points and IL-1β secretion in the culture supernatants was measured by ELISA. **D**, **E** The cells were incubated with indicated stimuli for 6 h. Pro- or active forms of caspase-1 and IL-1β in the culture supernatants (Sup) and pro-IL-1β and β-actin in the cell lysates (Cell) were detected by immunoblotting. **F**, **G** The cells were incubated with indicated stimuli for 6 h. IL-1β expression in the culture supernatants was measured by ELISA. Pam3CSK4 was used as a positive control. All results are expressed as mean ± SD of triplicate samples. *p < 0.05.

We first examined the effect of SCFAs on Ef.LTA-induced inflammasome activation. PMA-differentiated THP-1 cells were treated with Ef.LTA in the presence or absence of acetate, butyrate, or propionate. The degree of inflammasome activation was determined by the detection of cleaved caspase-1 (p20) and mature IL-1β (p17) in culture supernatants. While Ef.LTA alone induced IL-1β at negligible levels, Ef.LTA in combination with butyrate (highly) or propionate (moderately) induced IL-1β expression (Fig. 1A). Ef.LTA alone induced pro-IL-1β expression but not mature IL-1β secretion, indicating that Ef.LTA primes the cells to generate pro-IL-1β. Ef.LTA in combination with butyrate or propionate induced both pro-IL1β expression and mature IL-1β secretion (Fig. 1B). Cleaved caspase-1 was not detected in the group treated with Ef.LTA only. However, when the cells were co-treated with SCFAs and Ef.LTA, both mature IL-1β and cleaved caspase-1 were notably detected in the cell culture supernatants. In addition, butyrate (highly) and propionate (moderately) enhanced mature IL-1β secretion in the presence of Ef.LTA while acetate did not show an effect. Caspase-1 activation was also observed in the culture supernatant from the cells treated with Ef.LTA plus either butyrate or propionate. Among the SCFAs examined, butyrate most potently induced Ef.LTA-induced inflammasome activation. The expression of IL-1β in the cells co-treated with Ef.LTA and butyrate was increased in a time-dependent manner (Fig. 1C). In addition, Ef.LTA induced caspase-1 activation and IL-1β secretion in the presence of butyrate in a dose-dependent manner (Fig. 1D, F). Butyrate also induced caspase-1 activation and IL-1β secretion in the presence of Ef.LTA in a dose-dependent manner (Fig. 1E, G). These results suggest that caspase-1-mediated IL-1β maturation is related with the Ef.LTA/butyrate-activated inflammasome in PMA-differentiated THP-1 cells.

### D-alanine moieties of Ef.LTA structure is critical for the inflammasome activation

**Fig. 2:**
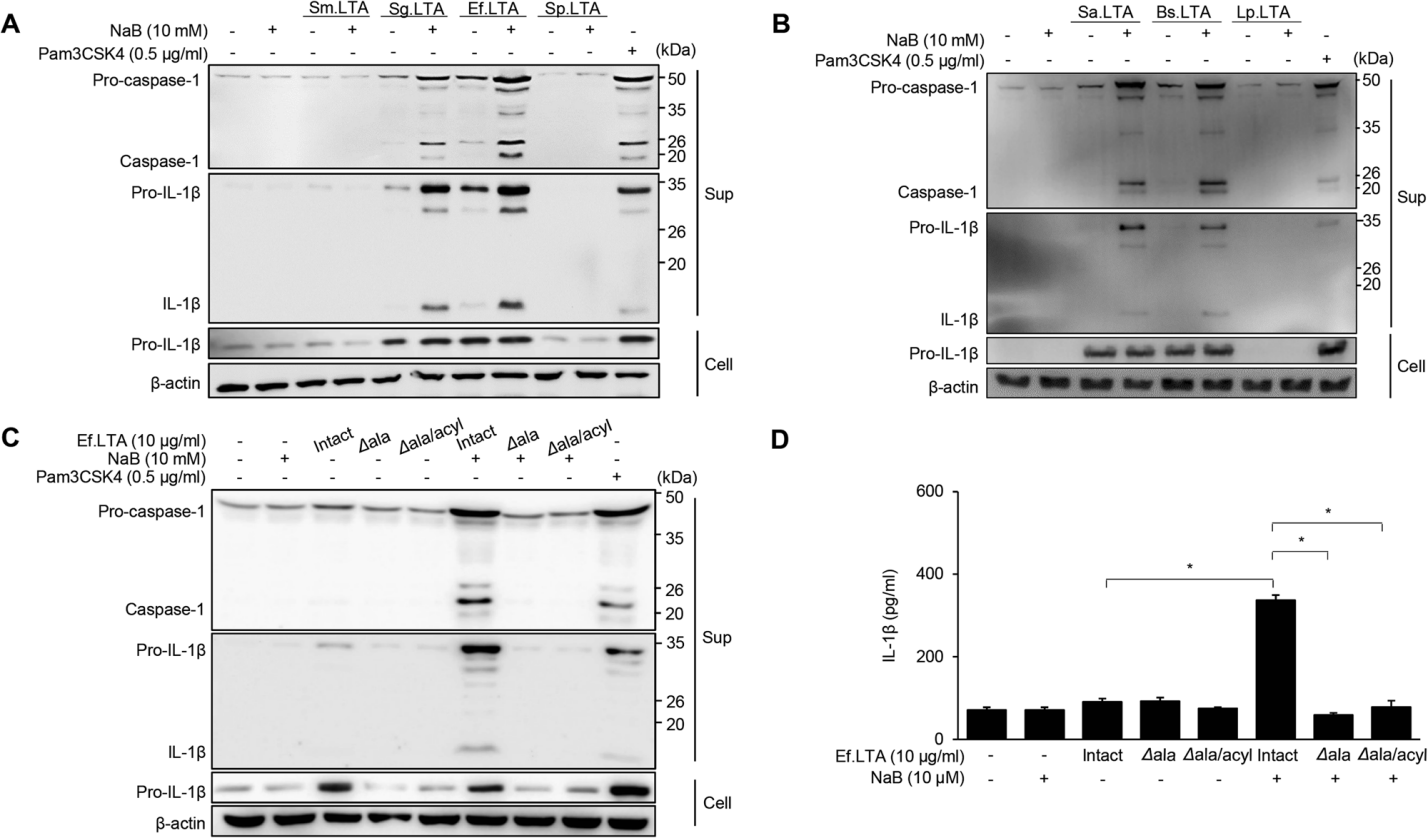
D-alanine moieties of Ef.LTA are critical for inflammasome activation. **A**, **B** PMA-differentiated THP-1 cells were stimulated with 10 μg/ml of LTAs purified from *Streptococcus mutans* (Sm.LTA), *Streptococcus gordonii* (Sg.LTA), *E. faecalis* (Ef.LTA), *Streptococcus pneumoniae* (Sp.LTA), *Staphylococcus aureus* (Sa.LTA), *Bacillus subtilis* (Bs.LTA) and *Lactobacillus plantarum* (Lp.LTA) or 0.5 μg/ml of Pam3CSK4 in the presence or absence of NaB (10 mM) for 6 h. After the stimulation, the pro- or active forms of caspase-1 and IL-1β in the culture supernatants (Sup) and pro-IL-1β and β-actin in the cell lysates (Cell) were detected by immunoblotting. **C**, **D** PMA-differentiated THP-1 cells were stimulated with dealanylated (*Δ*ala)-Ef.LTA and dealanylated/deacylated (*Δ*ala/acyl)-Ef.LTA in the presence or absence of NaB for 6 h. **C** Pro- or active forms of caspase-1 and IL-1β in the culture supernatants (Sup) and pro-IL-1β and β-actin in the cell lysates (Cell) were detected by immunoblotting. **D** IL-1β expression in the culture supernatants was measured by ELISA. Pam3CSK4 was used as a positive control. The results shown are representative of triplicate experiments.

Next, to determine whether this is a common phenomenon associated with LTAs of Gram-positive bacteria or due to the unique characteristics of Ef.LTA, we examined the inflammasome activation by LTAs purified from other Gram-positive bacteria in the presence of butyrate. As shown in Fig. 2A, B, LTAs from *S. gordonii*, *E. faecalis*, *S. aureus*, and *B. subtilis* efficiently induced caspase-1 activation and IL-1β secretion in the presence of butyrate while LTAs from *S. mutans, S. pneumoniae*, and *L. plantarum* did not show these effects. We previously reported that the D-alanine and glycolipid moieties of LTA are responsible for its immunological properties [25–27]. When the cells were stimulated with dealanylated-Ef.LTA and dealanylated/deacylated-Ef.LTA in the presence or absence of butyrate, neither dealanylated- nor dealanylated/deacylated-Ef.LTA induced caspase-1 activation and IL-1β secretion in the presence of butyrate (Fig. 2C, D). These results suggest that D-alanine moieties of LTA are important for Ef.LTA/butyrate inflammasome activation in macrophages.

### Ef.LTA/butyrate induces NLRP3 inflammasome activation

**Fig. 3:**
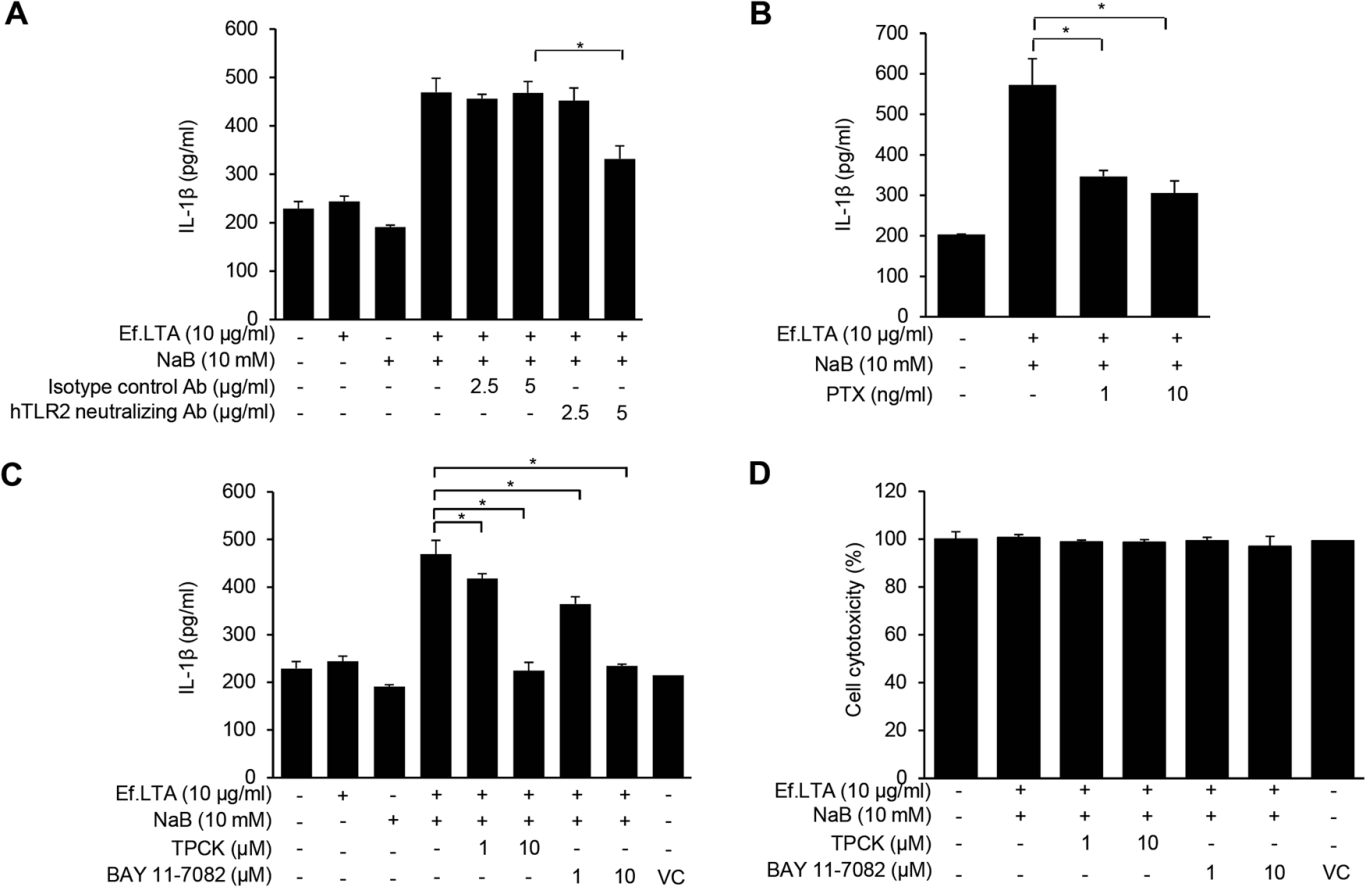
TLR2/GPCR are crucial for Ef.LTA/NaB-induced inflammasome activation. PMA-differentiated THP-1 cells were pre-treated with the TLR2-neutralizing antibody or its isotype control antibody (**A**), PTX (**B**), TPCK, or BAY 11-7082 (**C**) for 1 h and subsequently co-stimulated with Ef.LTA (10 μg/ml) and NaB (10 mM) for an additional 6 h. IL-1β expression in the culture supernatants was measured by ELISA. *p* < 0.05. **D** The cells were subjected to MTT assay to determine cell viability. VC denotes vehicle control (DMSO).

**Fig. 4:**
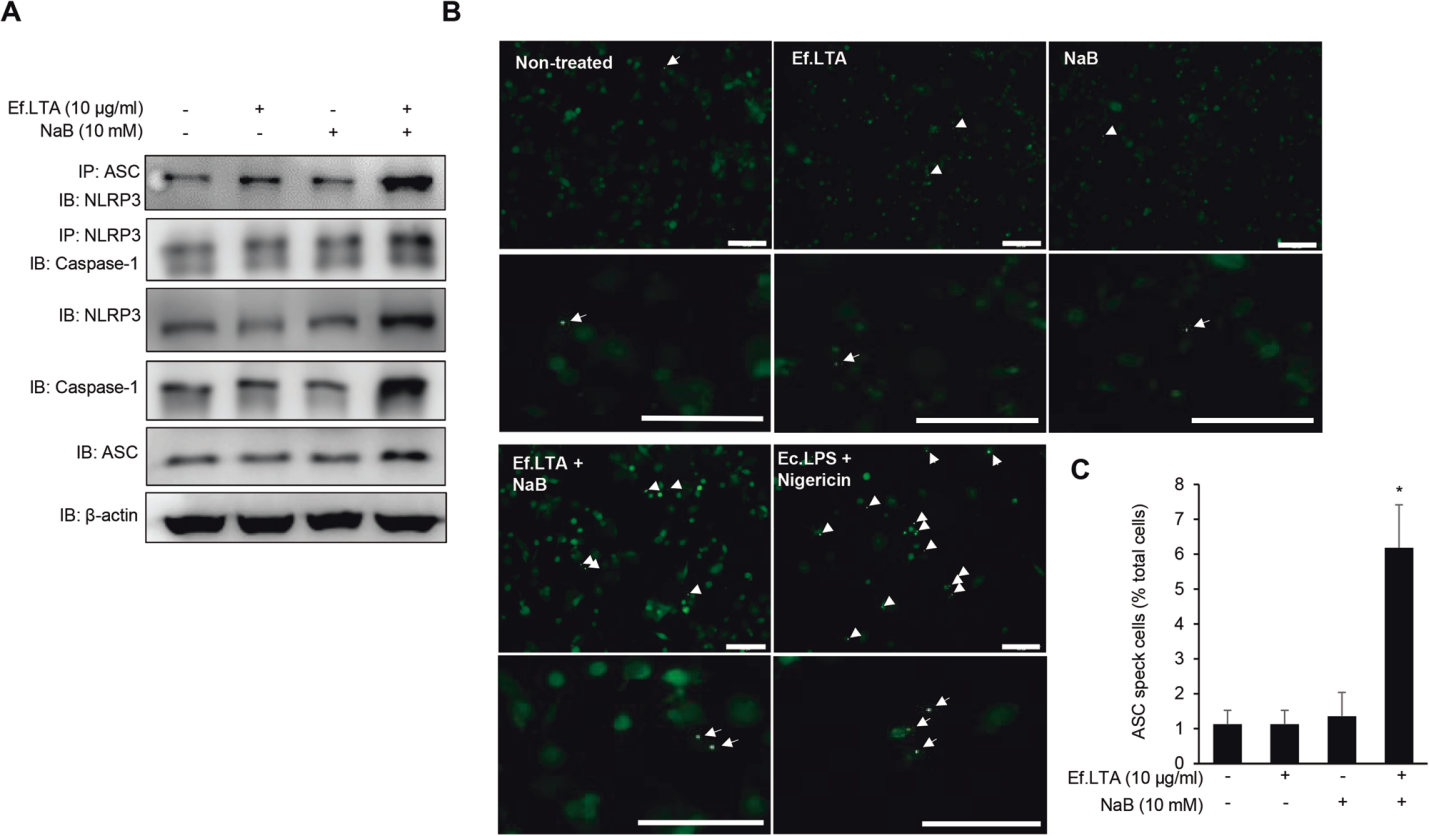
Ef.LTA/NaB activates the NLRP3 inflammasome. **A** PMA-differentiated THP-1 cells were treated with NaB (10 mM) in the presence or absence of Ef.LTA (10 μg/ml) for 6 h. The cells were lysed, and ASC or NLRP3 proteins were immunoprecipitated with appropriate antibodies. Then, the immunoprecipitated complexes were analyzed by Western blotting using specific antibodies to NLRP3 or caspase-1. The expression of NLRP3, caspase-1, and ASC was determined by Western blotting. **B**, **C** PMA-differentiated ASC-GFP-THP-1 cells were treated with Ef.LTA (10 μg/ml), NaB (10 μM), or Ef.LTA/NaB for 6 h. **B** The cells were fixed, observed and photographed by fluorescence microscopy. The arrows indicate ASC speck formations. **C** ASC specks per cell were enumerated using ImageJ software. *E. coli* LPS (1 μg/ml)/Nigericin (10 mM) was used as a positive control. *p < 0.05.

NLRP3 inflammasome is necessary for caspase-1 activation and IL-1β production [28]. One suggestion is that a microbial component-mediated priming signal stimulates the production of NLRP3 and pro-IL-1β through nuclear factor kappa-B (NF-κB) [29]. In addition, G-protein coupled receptor (GPCR) and toll-like receptor 2 (TLR2) sense SCFAs and Ef.LTA, respectively [4, 30]. Thus, we first examined whether TLR2 and GPCR were related to Ef.LTA/butyrate-induced IL-1β expression. When the cells were pre-treated with TLR2-neutralizing antibody or PTX, an inhibitor of GPCR, Ef.LTA/butyrate-induced IL-1β expression was significantly reduced (Fig. 3A, B). The cells pre-treated with NF-κB inhibitors such as BAY 11-7082 and TPCK inhibited Ef.LTA/butyrate-induced IL-1β expression without affecting cell viability (Fig. 3C, D). These results suggest that TLR2/GPCR recognition of Ef.LTA/butyrate and NF-κB activation play critical roles in IL-1β expression. The NLRP3 inflammasome complex consisting of ASC and pro-caspase-1 promotes IL-1β secretion [31]. Indeed, the cells treated with Ef.LTA/butyrate showed an increase in the expression of NLRP3, ASC, and caspase-1 and the colocalization of NLRP3 with ASC or caspase-1 (Fig. 4A). Ef.LTA/butyrate but not Ef.LTA or butyrate alone also induced ASC speck formation (Fig. 4B, C). These results suggest that Ef.LTA/butyrate, but not Ef.LTA or butyrate alone, promotes NLRP3 inflammasome activation by forming the complex consisting of NLRP3, ASC, and caspase-1.

### Ef.LTA/butyrate induces IL-1β secretion via potassium (K^+^) efflux

**Fig. 5:**
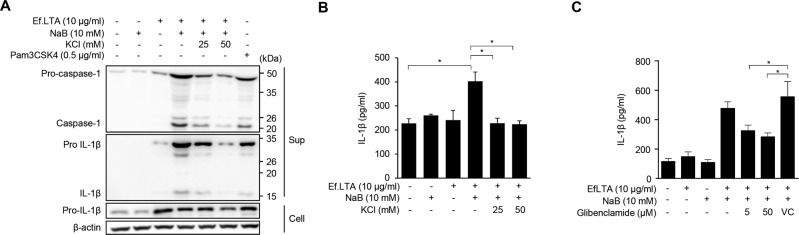
K^+^ efflux is crucial for Ef.LTA/NaB-induced inflammasome activation. **A** PMA-differentiated THP-1 cells were pre-treated with or without 25 or 50 mM of potassium chloride (KCl) for 1 h and subsequently co-stimulated with Ef.LTA (10 μg/ml) and NaB (10 mM) for 6 h. Then, the culture supernatants were collected and the levels of pro- or active caspase-1 and IL-1β were determined by immunoblotting. **B** The concentrations of IL-1β secreted into the culture supernatants were measured using ELISA. **C** The cells were pre-treated with glibenclamide (5 or 50 μM) for 1 h and subsequently co-stimulated with Ef.LTA (10 μg/ml) and NaB (10 mM) for an additional 6 h. IL-1β expression in the culture supernatants was measured by ELISA. Pam3CSK4 was used as a positive control. VC denotes vehicle control (DMSO). *p < 0.05. The results shown are representative of triplicate experiments.

Blocking of K^+^ efflux by extracellular potassium reduces IL-1β secretion by attenuating NLRP3 activation [32]. Therefore, we hypothesized that K^+^ efflux is critical for Ef.LTA/butyrate-induced IL-1β secretion. As a result, high extracellular KCl suppressed Ef.LTA/butyrate-induced caspase-1 activation and IL-1β production (Fig. 5A, B). Consistently, glibenclamide, an ATP-sensitive K^+^ channel blocker, inhibited IL-1β induced by Ef.LTA/butyrate (Fig. 5C). These findings imply that Ef.LTA/butyrate-induced NLRP3 inflammasome formation and IL-1β release are dependent on K^+^ efflux.

### Activation of both caspase-1 and caspase-4 is required for Ef.LTA/butyrate-induced inflammasome activation

**Fig. 6:**
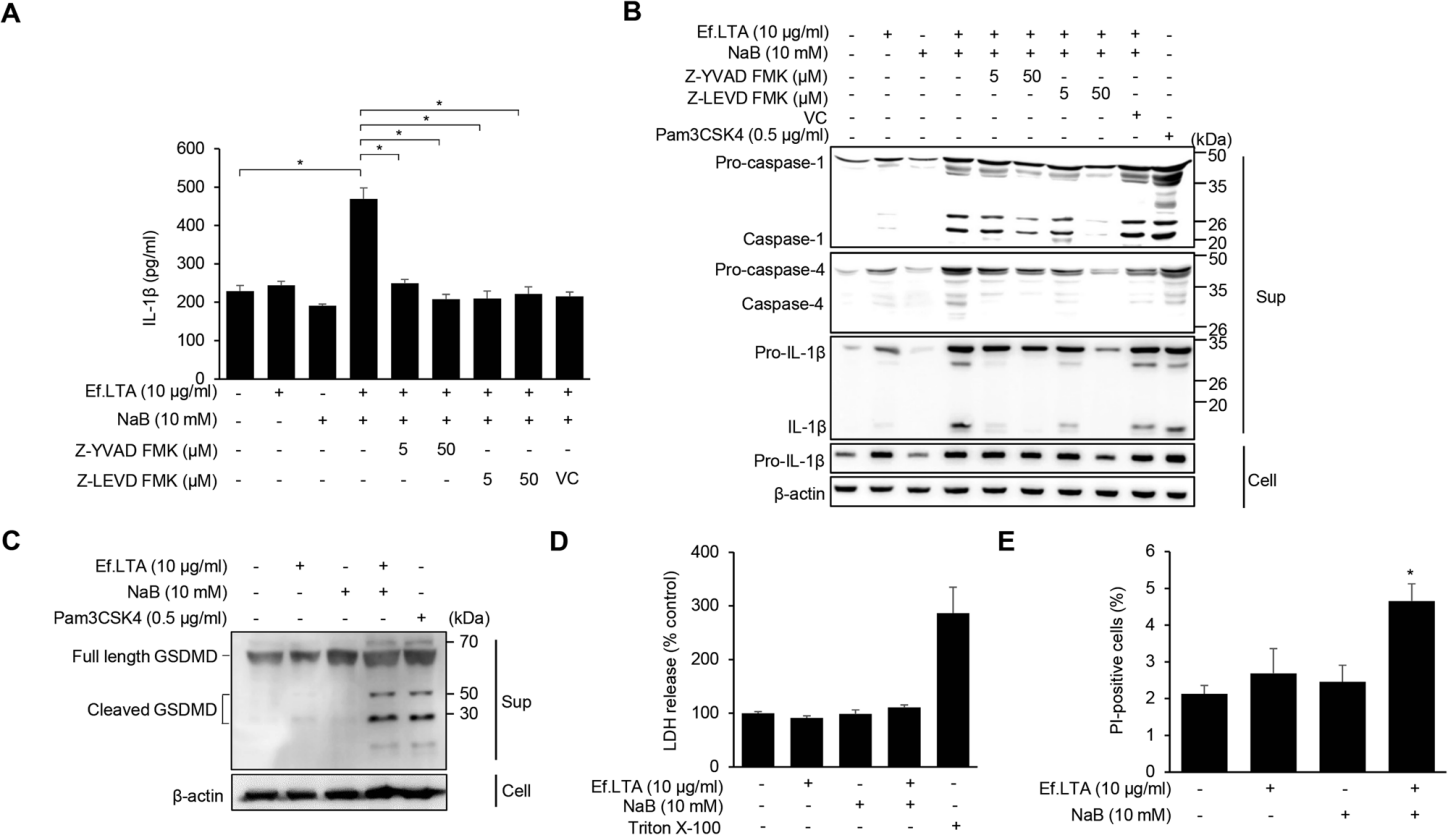
Activation of both caspase-1 and caspase-4 is required for Ef.LTA/NaB-induced inflammasome activation. **A** PMA-differentiated THP-1 cells were pre-treated with the indicated concentrations of the caspase-1 inhibitor (Z-YVAD-FMK), the caspase-4 inhibitor (Z-LEVD-FMK), for 1 h and subsequently co-stimulated with Ef.LTA (10 μg/ml) and NaB (10 mM) for an additional 6 h. IL-1β expression in the culture supernatants was measured by ELISA. **B** Pro- or active caspase-1 and IL-1β in the culture supernatants (Sup) and pro-IL-1β and β-actin in the cell lysates (Cell) were detected by immunoblotting. Pam3CSK4 was used as a positive control. **C** The cells were co-stimulated with Ef.LTA (10 μg/ml) and NaB (10 mM) for 6 h. Then, the culture supernatants were collected and the level of cleaved GSDMD was determined by immunoblotting. **D** The cells were co-stimulated with Ef.LTA (10 μg/ml) and NaB (10 mM) for 24 h. The LDH in culture supernatants was measured using the LDH-cytotoxicity colorimetric assay kit. **E** The cells were co-stimulated with Ef.LTA (10 μg/ml) and NaB (10 mM) for 6 h and stained with PI. The images were captured under a microscope and PI-positive cells were counted by ImageJ software. VC denotes vehicle control (DMSO). The results shown are representative of triplicate experiments. All results are expressed as mean ± SD of triplicate samples. *p < 0.05.

Inflammasomes are activated through canonical and non-canonical pathways mediated through caspase-1 and caspase-4, respectively [33]. When the cells were pre-treated with inhibitors for caspase-1 (Z-YVAD FMK) or caspase-4 (Z-LEVD FMK), IL-1β secretion by Ef.LTA/butyrate was inhibited (Fig. 6A, B), implying that both canonical and non-canonical pathways are involved. Active caspase-1 and caspase-4/caspase-5/caspase-11 have been known to cleave Gasdermin D (GSDMD), and the N-terminal domain of GSDMD induces cytokine release and pyroptosis via membrane pore formation [34, 35]. Therefore, we examined whether GSDMD is cleaved by Ef.LTA/butyrate. As shown in Fig. 6C, Ef.LTA/butyrate, but not Ef.LTA or butyrate alone, increased cleavage of GSDMD. However, Ef.LTA/butyrate did not induce LDH release, a marker of pyroptosis (Fig. 6D). Ef.LTA/butyrate significantly increased the number of PI-positive cells, but the absolute percentage of PI-positive cells was markedly low (<5%) (Fig. 6E). These results indicate that Ef.LTA/butyrate induce GSDMD-dependent IL-1β secretion without pyroptosis.

### Histone deacetylase (HDAC) inhibition modulates the Ef.LTA-induced IL-1β secretion via caspase-1 activation

**Fig. 7:**
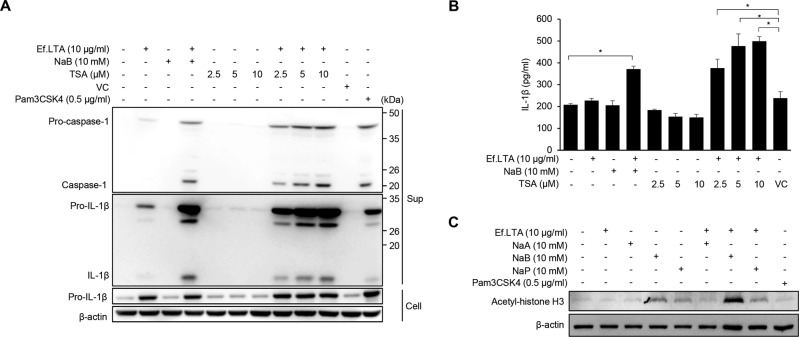
HDAC inhibition is necessary for inflammasome activation by Ef.LTA/NaB. **A** PMA-differentiated THP-1 cells were stimulated with Ef.LTA (10 μg/ml) in the presence of NaB (10 mM) or indicated concentrations of trichostatin A (TSA) for 6 h. Then, the culture supernatants were collected and the levels of pro- or active caspase-1, IL-1β were determined by immunoblotting. **B** The concentrations of IL-1β secreted into the culture supernatants was measured using ELISA. The results shown are representative of triplicate experiments. Pam3CSK4 was used as a positive control. VC denotes vehicle control (DMSO). All results are expressed as mean ± SD of triplicate samples. *p < 0.05. **C** The cells were stimulated with NaB, NaB, or NaP in the presence or absence of Ef.LTA. The cells were lysed and subjected to Western blotting using anti-acetyl-histone H3 antibody.

Butyrate acts as an HDAC inhibitor that modulates gene expression through histone acetylation [36]. HDAC inhibitors increase LPS-induced IL-1β expression in macrophages and dendritic cells [37]. Thus, we examined whether HDAC inhibition is related to Ef.LTA-induced IL-1β secretion. Like Ef.LTA/butyrate, Ef.LTA and trichostatin A (an HDAC inhibitor) co-treatment efficiently induced secretion of mature IL-1β and caspase-1 activation (Fig. 7A, B). In addition, Ef.LTA/butyrate increased histone acetylation (Fig. 7C). Therefore, HDAC inhibition might be involved in Ef.LTA/butyrate-induced inflammasome formation.

### Ef.LTA and butyrate co-treatment increases IL-1β expression in rat apical area

**Fig. 8:**
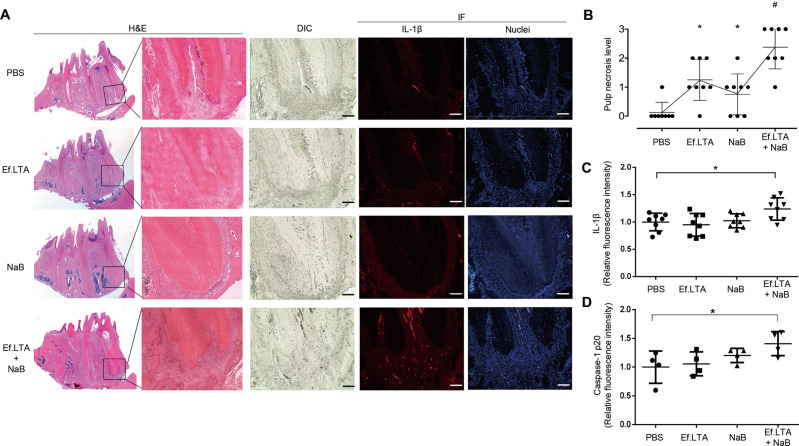
Ef.LTA together with NaB cooperatively increases IL-1β expression around the root apex in rat apical periodontitis model. Six-week-old female pathogen-free Sprague-Dawley rats were anesthetized and the pulp of bilateral first mandibular molars was exposed. A collagen sheet soaked with PBS, 10 μg of Ef.LTA, 10 mM of NaB, or Ef.LTA/NaB was inserted into the pulp chamber and then plugged with a sterile cotton pellet. **A** After 4 weeks, the mandibles were fixed and decalcified. Decalcified mandibles were embedded in paraffin, sectioned longitudinally and attached to glass slides. The sections of the mandibular molars were stained with H&E, and the expression of IL-1β was identified by immunofluorescence staining using anti-rat IL-1β antibody. Nuclei were stained with Hoechst 33258. The samples were observed using fluorescence microscopy. DIC = differential interference contrast. Scale bars = 200 μm. **B** The pulp necrosis level was assessed histometrically for the root canal of each tooth. Score 0 indicates no pulp cell necrosis in the entire root. Scores of 1, 2, and 3 indicate partial necrosis in the coronal one-third of the root canal, necrosis in two-thirds of the whole root canal, and full necrosis of the root canal, respectively. **C**, **D** Quantitative analysis of the relative fluorescence intensity was performed using ZEN software. *p < 0.05.

*E. faecalis* and butyrate are commonly found in the root canals of teeth with refractory apical periodontitis [14, 38]. Thus, we examined the effect of Ef.LTA and butyrate on IL-1β expression in vivo using the previously established rat apical periodontitis model [24]. As shown in Fig. 8A, in PBS group, H&E or immunofluorescence staining of mandibular molar tissue sections showed a tissue damage limited to the coronal pulp chamber. The pulp tissue in the root canal remained viable with increased fibrosis, neutrophil aggregation, and vascularity in the upper part of the root canal. In the Ef.LTA treatment group, some specimens had areas of inflammatory necrosis extending to the middle root area with viable pulp tissue remaining underneath. In the Ef.LTA and butyrate co-treatment group, necrosis of pulp tissue was found in the entire pulp area including root canals, and neutrophil aggregation and bone resorption area was relatively large around the root apex (Fig. 8A). The pulp necrosis level in the Ef.LTA and butyrate co-treatment group was higher than the other groups (Fig. 8B). We also observed that the teeth treated with Ef.LTA and butyrate exhibited a markedly increased IL-1β and caspase-1 p20 expression around the root apex (Fig. 8C, D). These results provide in vivo evidence for pathogenesis of apical periodontitis and Ef.LTA/butyrate-induced IL-1β production.

## Discussion

As summarized in Fig. S1, Ef.LTA in combination with butyrate potently enhanced IL-1β expression and inflammasome activation involving NLRP3, ASC, and caspase-1 through both canonical and non-canonical pathways. Recognition of Ef.LTA/butyrate by TLR2/GPCR, K^+^ efflux, and GSDMD cleavage are critical for Ef.LTA/butyrate-induced IL-1β expression. The increased IL-1β and caspase-1 expression by Ef.LTA/butyrate was confirmed in the rat apical periodontitis model. Therefore, we suggest that Ef.LTA/butyrate-enhanced inflammasome activation might be important for the inflammation of apical lesions that contributes to the development of apical periodontitis.

SCFAs and Ef.LTA synergistically induced the inflammasome activation and IL-1β expression in macrophages and rat apical periodontitis models. Ef.LTA primes the cells to induce pro-IL-1β, and NaB promotes IL-1β secretion via caspase-1 expression and activation through K^+^ efflux, the formation of NLRP3 inflammasome complex, and HDAC inhibition. Consistent with these findings, SCFAs including acetate, butyrate, and propionate enhanced inflammasome activation and IL-1β secretion in macrophages infected with *Salmonella enterica* serovar Typhimurium [39]. In contrast, we showed that LTA alone was insufficient to induce inflammasome activation. Concordantly, Ef.LTA was reported to directly activate the NLRP3 inflammasome, but Ef.LTA-induced IL-1β was extremely low (less than 15 pg/ml) and caspase-1 expression was observed only in the cell lysate but not in the culture supernatant [40]. Although some studies reported that butyrate inhibits NLRP3 inflammasome [41, 42], some evidence supported positive correlation between butyrate and inflammasome activation in LPS-primed monocytes or macrophages [11, 39].

Both TLR2 and GPCR appear to be essential for Ef.LTA/butyrate-induced inflammasome activation. Indeed, the binding of LTA to TLR2 activates inflammatory responses [4]. For instance, LTA-induced inflammatory mediators and chemokines were not observed in TLR2-deficient macrophages [5, 43], suggesting that TLR2 is pivotal for LTA-induced inflammatory responses. GPCRs including GPR43, GPR41, and GPR109A are essential for SCFA-mediated regulation [44]. In addition, TLRs have been shown to modulate GPCR-mediated innate immune responses through regulation of the expression of arrestin-/GPCR-associated kinases [45]. Therefore, the inhibition of TLR2/GPCR would be a target to control Gram-positive bacteria-induced inflammatory responses.

In our study, both caspase-1 (canonical inflammasome) and caspase-4 (non-canonical inflammasome) are required for Ef.LTA/butyrate-induced IL-β release. Ef.LTA/butyrate, but not Ef.LTA alone, efficiently activated the NLRP3 inflammasome. Ef.LTA/butyrate increased the expression of NLRP3, ASC, and caspase-1 and the colocalization of NLRP3 with ASC or caspase-1. SCFAs have been reported to bind to ASC and NLRP3 [39], yet the regulatory mechanism of caspase-1 expression by SCFAs is not clear and needs further study. Caspase-4 is related to Ef.LTA/butyrate-induced IL-1β, but the mechanism for caspase-4 activation also needs further study. Cytosolic *S. aureus* LTA induces caspase-1 activation and IL-1β/IL-18 secretion through NLRP6/caspase-11 activation in macrophages [23]. This suggests that the inflammasome activation by direct binding of cytosolic LTA to NLRP6 contributes to the progression of intracellular infection by *Listeria monocytogenes*. In addition, cytosolic *S. mutans* LTA induces IL-1β secretion via the NLRP6-caspase 4 inflammasome in LPS-primed human dental pulp cells [46]. We also observed that Ef.LTA delivered intracellularly significantly increases IL-1β production (Fig. S2). Further studies are required to determine the differences in the regulatory mechanisms of NLRP6/caspase-11-mediated inflammasome activation between extracellular and intracellular pathogens.

We observed that LTAs of some bacterial species, but not all, could synergistically induce inflammasome activation when co-treated with butyrate. The differential immunostimulating activity of Gram-positive bacteria may be due to the difference in their LTA structure [6]. LTA can be classified into five types based on molecular structure [4]. The number of repeating units and D-alanine contents and the degree of the saturation of the acyl chain in LTA are different for each bacterial species [47, 48]. LTAs from *S. aureus* and *S. pneumoniae* induce inflammatory responses through the production of nitric oxide and IL-6 [49, 50]. However, *Lactobacilli* LTAs have anti-inflammatory properties. For instance, *L. plantarum* LTA attenuates Pam2CSK4 and Poly I:C-induced IL-8 production in intestinal epithelial cells [27, 51]. The acyl group of *S. aureus* LTA and *L. rhamnosus* GG LTA contains saturated and unsaturated fatty acid, respectively [48]. We also observed in the present study that neither dealanylated nor dealanylated/deacylated Ef.LTA induced inflammasome activation in the presence of butyrate. These results indicate that the structure of LTA may be a critical factor in determining inflammasome activation in the presence of SCFAs.

We showed that Ef.LTA/butyrate efficiently induced IL-1β in PMA-differentiated THP-1 cells and apical lesions in rats with apical periodontitis. Consistent with our results, butyrate potentiated LPS-induced IL-1β secretion from THP-1 cells [11]. SCFAs increased TNF-α-induced IL-6 and CXCL8 in human lung fibroblasts [12]. Butyrate increased a killed *Vibrio cholerae*-induced CCL20 secretion from human intestinal epithelial cells [52]. In addition, orally administered SCFAs could induce renal disease via increased inflammatory Th17 and Th1 cells in ureter and kidney tissues in mice [53]. However, an impact of SCFAs on anti-inflammatory responses has also been reported. SCFAs reduced pro-inflammatory molecules such as nitric oxide and TNF-α induced by staphylococcal lipoproteins or *E. coli* LPS in murine macrophages and human peripheral blood mononuclear cells [54, 55]. SCFAs reduced the risk of LPS- and pathogen-induced diseases such as periodontitis and sepsis through down-regulation of pro-inflammatory cytokines [56, 57]. A reduction of butyrate-producing bacteria such as *Roseburia hominis* and *Faecalibacterium prausnitzii* is related with ulcerative colitis [58]. The opposing effects of SCFAs on inflammatory responses may be due to tissue tropism or differential effects on local vs. systemic inflammation.

Inflammasome activation plays an important role in the pathogenesis of apical periodontitis. For instance, periapical tissue sections exhibited increased expression of NLRP3 proteins [22]. Increased expression of NLRP3, caspase-1, and IL-1β in periapical inflamed tissue was also reported [40]. In this study, we also observed the positive correlation between Ef.LTA/butyrate co-treatment and increased IL-1β expression around the root apex and pulp necrosis level. Ef.LTA/butyrate did not induce pyroptosis of THP-1 cells in our study. However, because Ef.LTA/butyrate induced pulp necrosis in vivo, further study is needed to elucidate the role of Ef.LTA/butyrate on dental pulp cell death. Collectively, IL-1β and the inflammasome might be used as molecular targets for apical periodontitis.

## Materials and methods

### Reagents and chemicals

*Bacillus subtilis* ATCC 6633, *E. faecalis* ATCC 29212, *Staphylococcus aureus* ATCC 29213, and *Streptococcus pneumoniae* ATCC 27336 were obtained from the American Type Culture Collection (ATCC, Manassas, VA, USA). *Streptococcus mutans* KCTC 3065 and *Lactobacillus plantarum* KCTC 10887BP were obtained from the Korean Collection for Type Culture (Daejeon, Korea). *Streptococcus gordonii* CH1 was kindly provided from Prof. Paul M. Sullam (University of California at San Francisco). LTAs were purified from *B. subtilis*, *E. faecalis*, *L. plantarum*, *S. aureus*, *S. gordonii*, *S. mutans*, and *S. pneumoniae* as previously described [6]. To remove the D-alanine of Ef.LTA, Ef.LTA was treated with 0.1 M Tris-HCl at pH 8.5 for 24 h. To remove D-alanine/acyl moieties, Ef.LTA was treated with 0.5 N NaOH for 2 h followed by neutralization of pH as previously described [25]. Pam3CSK4 was obtained from InvivoGen (San Diego, CA, USA). *Escherichia coli* LPS, dimethyl sulfoxide (DMSO), potassium chloride (KCl), phorbol 12-myristate 13-acetate (PMA), trichloroacetate (TCA), sodium acetate, sodium butyrate, sodium propionate, N-p-tosyl-L-phenylalanine chloromethyl ketone (TPCK), glibenclamide, and trichostatin A were purchased from Sigma-Aldrich Inc. (St. Louis, MO, USA). Z-YVAD-FMK and Z-LEVD-FMK were purchased from ApexBio (Hsinchu, Taiwan) and Biovision (Milpitas, CA, USA), respectively. Pertussis toxin (PTX) was purchased from Tocris Bioscience (Bristol, UK). Propidium iodide (PI) was obtained from BD Biosciences (San Jose, CA, USA). Antibodies are listed in Supplementary Table 1.

### Cell culture and stimulation

THP-1 cells were obtained from the ATCC and ASC-GFP-THP-1 cells were kindly provided from Prof. Je-Wook Yu (Yonsei University, Seoul, Korea). The cells were grown in RPMI 1640 medium containing 10% heat-inactivated fetal bovine serum (FBS) (Gibco, Burlington, ON, Canada), 100 U/ml penicillin, and 100 μg/ml streptomycin (Hyclone, Logan, UT, USA) at 37 °C in a humidified incubator with 5% CO_2_. THP-1 cells were differentiated into macrophages by treatment with 100 nM PMA for 2 days.

### Western blotting

PMA-differentiated THP-1 cells were stimulated with indicated stimuli for 6 h, and cells and culture supernatants were collected separately. The culture supernatants were mixed with 10% TCA at 4 °C for 30 min followed by centrifugation at 18,400 × *g* for 10 min. The precipitated cell pellets were washed with 100% acetone and mixed with 15 μl of 0.1 N NaOH. Western blotting was performed as previously described [5]. In a separate experiment, the cells were lysed and immunoprecipitated with anti-ASC or anti-NLRP3 antibody and protein G-agarose beads as previously described [59].

### Enzyme-linked immunosorbent assay (ELISA)

PMA-differentiated THP-1 cells were plated onto a 96-well culture plate overnight and stimulated with indicated stimuli. The commercial ELISA kit (Biolegend, San Diego, USA) was used to quantify IL-1β expression in the culture supernatants.

### ASC speck assay

ASC-GFP-THP-1 cells were seeded onto glass cover slips in 24-well culture plates at 1.25 × 10^5^ cells/well and cultured in the presence of 100 ng/ml of PMA for 48 h. Culture media were then replaced with fresh, serum-free RPMI 1640 media and cultured for additional 24 h. The cells were treated with Ef.LTA (10 μg/ml), NaB (10 μM), or Ef.LTA (10 μg/ml)/NaB (10 μM) for 6 h. *E. coli* LPS (1 μg/ml)/Nigericin (10 μM) was used as a positive control. At the end of the treatment, the cells were fixed, observed, and photographed by fluorescence microscopy. ASC specks per cell were enumerated using ImageJ software (NIH, Bethesda, MD, USA).

### PI staining

The cells were stained with 20 μΜ of PI and Hoechst 33258 for 20 min and the images were captured with a BX51 fluorescence microscopy with a DP72 digital camera (Olympus, Tokyo, Japan). PI-positive cells were counted by ImageJ. Five fields of cells (1000 cells) were counted and the data are presented as the percentage of PI-positive cells per total cells counted.

### Lactate dehydrogenase (LDH) release assay

PMA-differentiated THP-1 cells were plated onto a 96-well culture plate overnight and stimulated with indicated stimuli for 24 h. The presence of LDH in the cell culture supernatants was measured by LDH-cytotoxicity colorimetric assay kit (Biovision, Milpitas, CA, USA).

### Rat apical periodontitis model

The animal experiments were conducted under the approval of the Institutional Animal Care and Use Committee of Seoul National University (SNU-180607). The rat apical periodontitis model was prepared as described previously [24]. Six-week-old female pathogen-free Sprague-Dawley rats were purchased from Orient Bio (Seongnam, Korea). Pulp-exposed molars were randomly assigned into four groups according to the inserted agents. A collagen sheet soaked with PBS, 10 μg of Ef.LTA, 10 mM of NaB, or Ef.LTA plus NaB was inserted into the pulp chamber by slight plugging with a dental explorer tip for each group (*n* = 8/group). The remainder of the cavity in the pulp chamber was filled using dental adhesives and flowable composite resin (Single Bond Universal and Filtek Supreme Ultra Flowable; 3M ESPE, St. Paul, MN, USA). No sample was excluded in our analysis.

### Histology

The animals were euthanized 4 weeks after surgery, and half of the mandibles were fixed in 4% paraformaldehyde for 12 h. The mandibles were decalcified in 17% EDTA in PBS (pH 8.0) for 5 weeks at room temperature with agitation. Decalcified mandibles were embedded in paraffin, sectioned longitudinally, and attached on glass slides. The sections of the mandibular molars were stained with H&E or immunofluorescence staining, and the pulp necrosis level was assessed as previously described [24, 60]. The samples were assessed using BX51 fluorescence microscopy with a DP72 digital camera.

### Statistical analysis

The data are presented as mean values ± standard deviations (SD) from triplicate samples unless otherwise stated. Statistical significance was examined by one-way ANOVA, followed by Dunnett’s multiple comparisons test or Student’s *t* test using GraphPad Prism 6 software (GraphPad Software Inc., La Jolla, CA, USA).

## Supplementary information

Supplemental Figures and Table

Original Data File

## Data Availability

Data supporting the present study are available from the corresponding author upon request.

## References

[1] Graunaite I, Lodiene G, Maciulskiene V (2012). Pathogenesis of apical periodontitis: a literature review. J Oral Maxillofac Res.

[2] Nair PN (1997). Apical periodontitis: a dynamic encounter between root canal infection and host response. Periodontol 2000.

[3] Wang QQ, Zhang CF, Chu CH, Zhu XF (2012). Prevalence of *Enterococcus faecalis* in saliva and filled root canals of teeth associated with apical periodontitis. Int J Oral Sci.

[4] Kang SS, Sim JR, Yun CH, Han SH (2016). Lipoteichoic acids as a major virulence factor causing inflammatory responses via Toll-like receptor 2. Arch Pharm Res.

[5] Park OJ, Han JY, Baik JE, Jeon JH, Kang SS, Yun CH (2013). Lipoteichoic acid of *Enterococcus faecalis* induces the expression of chemokines via TLR2 and PAFR signaling pathways. J Leukoc Biol.

[6] Ryu YH, Baik JE, Yang JS, Kang SS, Im J, Yun CH (2009). Differential immunostimulatory effects of Gram-positive bacteria due to their lipoteichoic acids. Int Immunopharmacol.

[7] Park T, Im J, Kim AR, Lee D, Jeong S, Yun C-H (2021). Short-chain fatty acids inhibit the biofilm formation of *Streptococcus gordonii* through negative regulation of competence-stimulating peptide signaling pathway. J Microbiol.

[8] Koh A, De Vadder F, Kovatcheva-Datchary P, Backhed F (2016). From dietary fiber to host physiology: short-chain fatty acids as key bacterial metabolites. Cell..

[9] Rios-Covian D, Ruas-Madiedo P, Margolles A, Gueimonde M, de Los Reyes-Gavilan CG, Salazar N (2016). Intestinal short chain fatty acids and their link with diet and human health. Front Microbiol.

[10] Vinolo MA, Rodrigues HG, Hatanaka E, Sato FT, Sampaio SC, Curi R (2011). Suppressive effect of short-chain fatty acids on production of proinflammatory mediators by neutrophils. J Nutr Biochem.

[11] Ohira H, Fujioka Y, Katagiri C, Yano M, Mamoto R, Aoyama M (2012). Butyrate enhancement of inteleukin-1beta production via activation of oxidative stress pathways in lipopolysaccharide-stimulated THP-1 cells. J Clin Biochem Nutr.

[12] Rutting S, Xenaki D, Malouf M, Horvat JC, Wood LG, Hansbro PM (2019). Short-chain fatty acids increase TNFalpha-induced inflammation in primary human lung mesenchymal cells through the activation of p38 MAPK. Am J Physiol Lung Cell Mol Physiol.

[13] Niederman R, Buyle-Bodin Y, Lu BY, Robinson P, Naleway C (1997). Short-chain carboxylic acid concentration in human gingival crevicular fluid. J Dent Res.

[14] Provenzano JC, Rocas IN, Tavares LF, Neves BC, Siqueira JF (2015). Short-chain fatty acids in infected root canals of teeth with apical periodontitis before and after treatment. J Endod.

[15] Prochnicki T, Latz E (2017). Inflammasomes on the crossroads of innate immune recognition and metabolic control. Cell Metab.

[16] Guo H, Callaway JB, Ting JP (2015). Inflammasomes: mechanism of action, role in disease, and therapeutics. Nat Med.

[17] Swanson KV, Deng M, Ting JP (2019). The NLRP3 inflammasome: molecular activation and regulation to therapeutics. Nat Rev Immunol.

[18] Shiratori T, Kyumoto-Nakamura Y, Kukita A, Uehara N, Zhang J, Koda K (2018). IL-1beta induces pathologically activated osteoclasts bearing extremely high levels of resorbing activity: a possible pathological subpopulation of osteoclasts, accompanied by suppressed expression of Kindlin-3 and Talin-1. J Immunol.

[19] Graves D (2008). Cytokines that promote periodontal tissue destruction. J Periodontol.

[20] Figueredo CMS, Ribeiro MSM, Fischer RG, Gustafsson A (1999). Increased interleukin-1beta concentration in gingival crevicular fluid as a characteristic of periodontitis. J Periodontol.

[21] Yang NY, Zhou Y, Zhao HY, Liu XY, Sun Z, Shang JJ (2018). Increased interleukin 1alpha and interleukin 1beta expression is involved in the progression of periapical lesions in primary teeth. BMC Oral Health.

[22] Ran S, Liu B, Gu S, Sun Z, Liang J (2017). Analysis of the expression of NLRP3 and AIM2 in periapical lesions with apical periodontitis and microbial analysis outside the apical segment of teeth. Arch Oral Biol.

[23] Hara H, Seregin SS, Yang D, Fukase K, Chamaillard M, Alnemri ES (2018). The NLRP6 inflammasome recognizes lipoteichoic acid and regulates Gram-positive pathogen infection. Cell..

[24] Park OJ, Jeong MH, Lee EH, Cho MR, Hwang J, Cho S (2020). A pilot study of chronological microbiota changes in a rat apical periodontitis model. Microorganisms..

[25] Ahn KB, Baik JE, Park OJ, Yun CH, Han SH (2018). Lactobacillus plantarum lipoteichoic acid inhibits biofilm formation of Streptococcus mutans. PLoS ONE.

[26] Baik JE, Jang KS, Kang SS, Yun CH, Lee K, Kim BG (2011). Calcium hydroxide inactivates lipoteichoic acid from *Enterococcus faecalis* through deacylation of the lipid moiety. J Endod.

[27] Noh SY, Kang SS, Yun CH, Han SH (2015). Lipoteichoic acid from *Lactobacillus plantarum* inhibits Pam2CSK4-induced IL-8 production in human intestinal epithelial cells. Mol Immunol.

[28] Kelley N, Jeltema D, Duan Y, He Y (2019). The NLRP3 inflammasome: an overview of mechanisms of activation and regulation. Int J Mol Sci.

[29] He Y, Hara H, Nunez G (2016). Mechanism and regulation of NLRP3 inflammasome activation. Trends Biochem Sci.

[30] Sun M, Wu W, Liu Z, Cong Y (2017). Microbiota metabolite short chain fatty acids, GPCR, and inflammatory bowel diseases. J Gastroenterol.

[31] Yang Y, Wang H, Kouadir M, Song H, Shi F (2019). Recent advances in the mechanisms of NLRP3 inflammasome activation and its inhibitors. Cell Death Dis.

[32] Petrilli V, Papin S, Dostert C, Mayor A, Martinon F, Tschopp J (2007). Activation of the NALP3 inflammasome is triggered by low intracellular potassium concentration. Cell Death Differ.

[33] Casson CN, Yu J, Reyes VM, Taschuk FO, Yadav A, Copenhaver AM (2015). Human caspase-4 mediates noncanonical inflammasome activation against gram-negative bacterial pathogens. Proc Natl Acad Sci USA.

[34] Shi J, Gao W, Shao F (2017). Pyroptosis: gasdermin-mediated programmed necrotic cell death. Trends Biochem Sci.

[35] Broz P, Pelegrin P, Shao F (2020). The gasdermins, a protein family executing cell death and inflammation. Nat Rev Immunol.

[36] Rahman MM, Kukita A, Kukita T, Shobuike T, Nakamura T, Kohashi O (2003). Two histone deacetylase inhibitors, trichostatin A and sodium butyrate, suppress differentiation into osteoclasts but not into macrophages. Blood..

[37] Stammler D, Eigenbrod T, Menz S, Frick JS, Sweet MJ, Shakespear MR (2015). Inhibition of histone deacetylases permits lipopolysaccharide-mediated secretion of bioactive IL-1beta via a caspase-1-independent mechanism. J Immunol.

[38] Vidana R, Sullivan A, Billstrom H, Ahlquist M, Lund B (2011). *Enterococcus faecalis* infection in root canals—host-derived or exogenous source?. Lett Appl Microbiol.

[39] Tsugawa H, Kabe Y, Kanai A, Sugiura Y, Hida S, Taniguchi S (2020). Short-chain fatty acids bind to apoptosis-associated speck-like protein to activate inflammasome complex to prevent Salmonella infection. PLoS Biol.

[40] Wang L, Jin H, Ye D, Wang J, Ao X, Dong M (2016). *Enterococcus faecalis* lipoteichoic acid-induced NLRP3 inflammasome via the activation of the nuclear factor kappa B pathway. J Endod.

[41] Bian F, Xiao Y, Zaheer M, Volpe EA, Pflugfelder SC, Li DQ (2017). Inhibition of NLRP3 inflammasome pathway by butyrate improves corneal wound healing in corneal alkali burn. Int J Mol Sci.

[42] Wang X, He G, Peng Y, Zhong W, Wang Y, Zhang B (2015). Sodium butyrate alleviates adipocyte inflammation by inhibiting NLRP3 pathway. Sci Rep.

[43] Hong SW, Baik JE, Kang SS, Yun CH, Seo DG, Han SH (2014). Lipoteichoic acid of *Streptococcus mutans* interacts with Toll-like receptor 2 through the lipid moiety for induction of inflammatory mediators in murine macrophages. Mol Immunol.

[44] Yang G, Chen S, Deng B, Tan C, Deng J, Zhu G (2018). Implication of G protein-coupled receptor 43 in intestinal inflammation: a mini-review. Front Immunol.

[45] Loniewski K, Shi Y, Pestka J, Parameswaran N (2008). Toll-like receptors differentially regulate GPCR kinases and arrestins in primary macrophages. Mol Immunol.

[46] Tian XX, Li R, Liu C, Liu F, Yang LJ, Wang SP (2020). NLRP6-caspase 4 inflammasome activation in response to cariogenic bacterial lipoteichoic acid in human dental pulp inflammation. Int Endod J.

[47] Morath S, Geyer A, Spreitzer I, Hermann C, Hartung T (2002). Structural decomposition and heterogeneity of commercial lipoteichoic acid preparations. Infect Immun.

[48] Lebeer S, Claes IJ, Vanderleyden J (2012). Anti-inflammatory potential of probiotics: lipoteichoic acid makes a difference. Trends Microbiol.

[49] Han SH, Kim JH, Seo HS, Martin MH, Chung GH, Michalek SM (2006). Lipoteichoic acid-induced nitric oxide production depends on the activation of platelet-activating factor receptor and Jak2. J Immunol.

[50] Jeon JH, Kim SK, Baik JE, Kang SS, Yun CH, Chung DK (2012). Lipoteichoic acid of *Staphylococcus aureus* enhances IL-6 expression in activated human basophils. Comp Immunol Microbiol Infect Dis.

[51] Kim KW, Kang SS, Woo SJ, Park OJ, Ahn KB, Song KD (2017). Lipoteichoic acid of probiotic *Lactobacillus plantarum* attenuates Poly I:C-induced IL-8 production in porcine intestinal epithelial cells. Front Microbiol.

[52] Sim JR, Kang SS, Lee D, Yun CH, Han SH (2018). Killed whole-cell oral cholera vaccine induces CCL20 secretion by human intestinal epithelial cells in the presence of the short-chain fatty acid, butyrate. Front Immunol.

[53] Park J, Goergen CJ, HogenEsch H, Kim CH (2016). Chronically elevated levels of short-chain fatty acids induce T cell-mediated ureteritis and hydronephrosis. J Immunol.

[54] Park JW, Kim HY, Kim MG, Jeong S, Yun CH, Han SH (2019). Short-chain fatty acids inhibit Staphylococcal lipoprotein-induced nitric oxide production in murine macrophages. Immune Netw.

[55] Aoyama M, Kotani J, Usami M (2010). Butyrate and propionate induced activated or non-activated neutrophil apoptosis via HDAC inhibitor activity but without activating GPR-41/GPR-43 pathways. Nutrition..

[56] Xu M, Jiang Z, Wang C, Li N, Bo L, Zha Y (2019). Acetate attenuates inflammasome activation through GPR43-mediated Ca(2+)-dependent NLRP3 ubiquitination. Exp Mol Med.

[57] Wang F, Liu J, Weng T, Shen K, Chen Z, Yu Y (2017). The inflammation induced by lipopolysaccharide can be mitigated by short-chain fatty acid, butyrate, through upregulation of IL-10 in septic shock. Scand J Immunol.

[58] Machiels K, Joossens M, Sabino J, De Preter V, Arijs I, Eeckhaut V (2014). A decrease of the butyrate-producing species *Roseburia hominis* and *Faecalibacterium prausnitzii* defines dysbiosis in patients with ulcerative colitis. Gut..

[59] Park OJ, Kim HJ, Woo KM, Baek JH, Ryoo HM (2010). FGF2-activated ERK mitogen-activated protein kinase enhances Runx2 acetylation and stabilization. J Biol Chem.

[60] Song J, Jung KJ, Yang MJ, Han SC, Lee K (2021). Assessment of acute and repeated pulmonary toxicities of oligo(2-(2-ethoxy)ethoxyethyl guanidium chloride in mice. Toxicol Res.

