# Supplementary material for: Butyrate potentiates Enterococcus faecalis lipoteichoic acid-induced inflammasome activation via histone deacetylase inhibition
Source: Cell Death Discov. 2023 Mar 28;9:107. doi: 10.1038/s41420-023-01404-2 (PMC10050190; doi:10.1038/s41420-023-01404-2)
Supplement: Supplementary file 1 — Supplemental Figures and Table [file 41420_2023_1404_MOESM1_ESM.docx]

Supplementary Materials

# Supplementary Figures and Tables

## Supplementary Figures

**
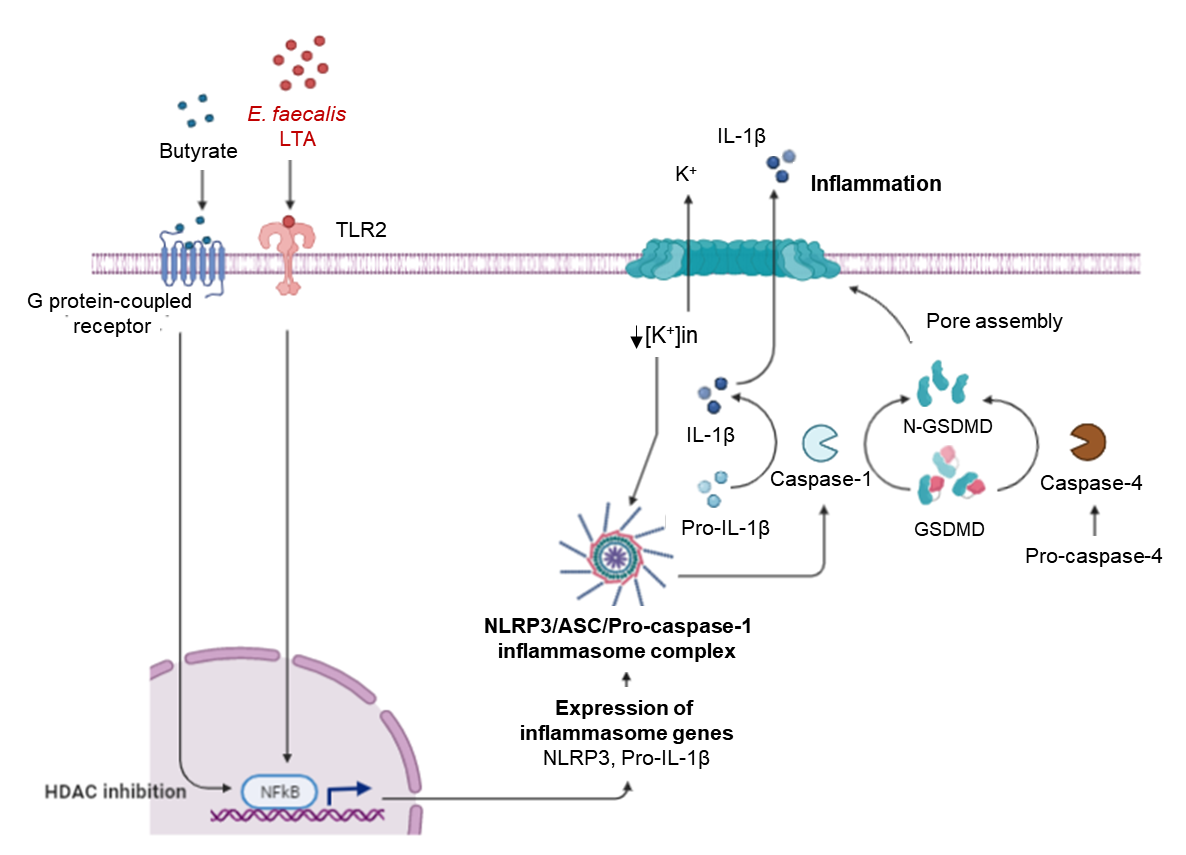
**

**Fig. S1.** **Proposed mechanism for inflammasome activation in macrophages treated with Ef.LTA and NaB.** TLR2 and GPCR are activated by Ef.LTA and NaB, respectively. This signaling promotes activation of NF-κB, leading to upregulation of NLRP3 and pro-IL-1β. Subsequently, Ef.LTA/NaB increases NLRP3/ASC/Pro-caspase-1 complex formation via K^+^ efflux, leading to the maturation and secretion of IL-1β. In addition, caspase-4 activation and HDAC inhibition are related with the Ef.LTA/NaB-induced inflammasome. Figure created with BioRender.com.


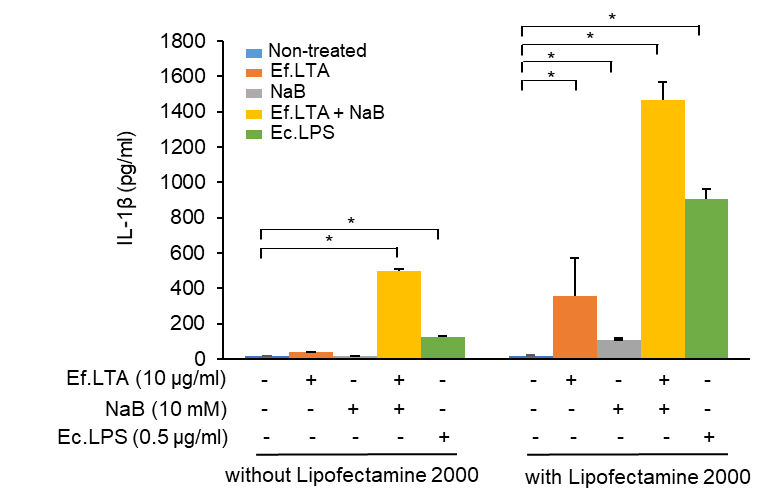


**Fig. S2.** **The intracellular Ef.LTA significantly increases IL-1β production.** PMA-differentiated THP-1 cells were stimulated with 10 μg/ml of Ef.LTA and/or 10 mM of NaB in the presence or absence of Lipofectamine 2000 for 6 h. LPS was used as positive control. IL-1β expression in culture supernatants was determined by ELISA.

## Supplementary Table

**Table S1. List of antibodies used in this study**

| Antibody or dye | Source | Catalog # | Use |
| --- | --- | --- | --- |
| Anti-caspase-1 | Cell Signaling Technology | 2225 | WB |
| Anti-caspase-4 | Cell Signaling Technology | 4450 | WB |
| Anti-acetyl-histone H3 (Lys9) | Cell Signaling Technology | 9649 | WB |
| Anti-Gasdermin D | Proteintech | 66387-1-Ig | WB |
| Anti-human IL-1β | Santa Cruz Biotechnology | sc-1250 | WB |
| Anti-ASC | BioLegend | 653902 | WB/IP |
| Anti-NLRP3 | Cell Signaling | 151015 | WB/IP |
| Anti-NLRP6 | Biorbyt | orb648291 | WB |
| Anti-β-actin | Santa Cruz Biotechnology | sc-47778 | WB |
| Anti-Mouse-HRP | Southern biotech | 1030-05 | WB |
| Anti-Rabbit-HRP | Southern biotech | 4010-05 | WB |
| Anti-rat IL-1β | Bio-Rad | AAR15G | IF |
| Anti-mouse caspase-1 p20 | Santa Cruz Biotechnology | SC-1218 | IF |
| Hoechst 33258 | Invitrogen | H21491 | IF |
| Anti-rabbit IgG Alexa 568 | Abcam | Ab175471 | IF |

WB: Western Blot; IF: Immunofluorescence; IP: Immunoprecipitation
