# Supplementary material for: Butyrate potentiates Enterococcus faecalis lipoteichoic acid-induced inflammasome activation via histone deacetylase inhibition
Source: Cell Death Discov. 2023 Mar 28;9:107. doi: 10.1038/s41420-023-01404-2 (PMC10050190; doi:10.1038/s41420-023-01404-2)
Supplement: Supplementary file 2 — Original Data File [file 41420_2023_1404_MOESM2_ESM.pptx]

## Slide 1
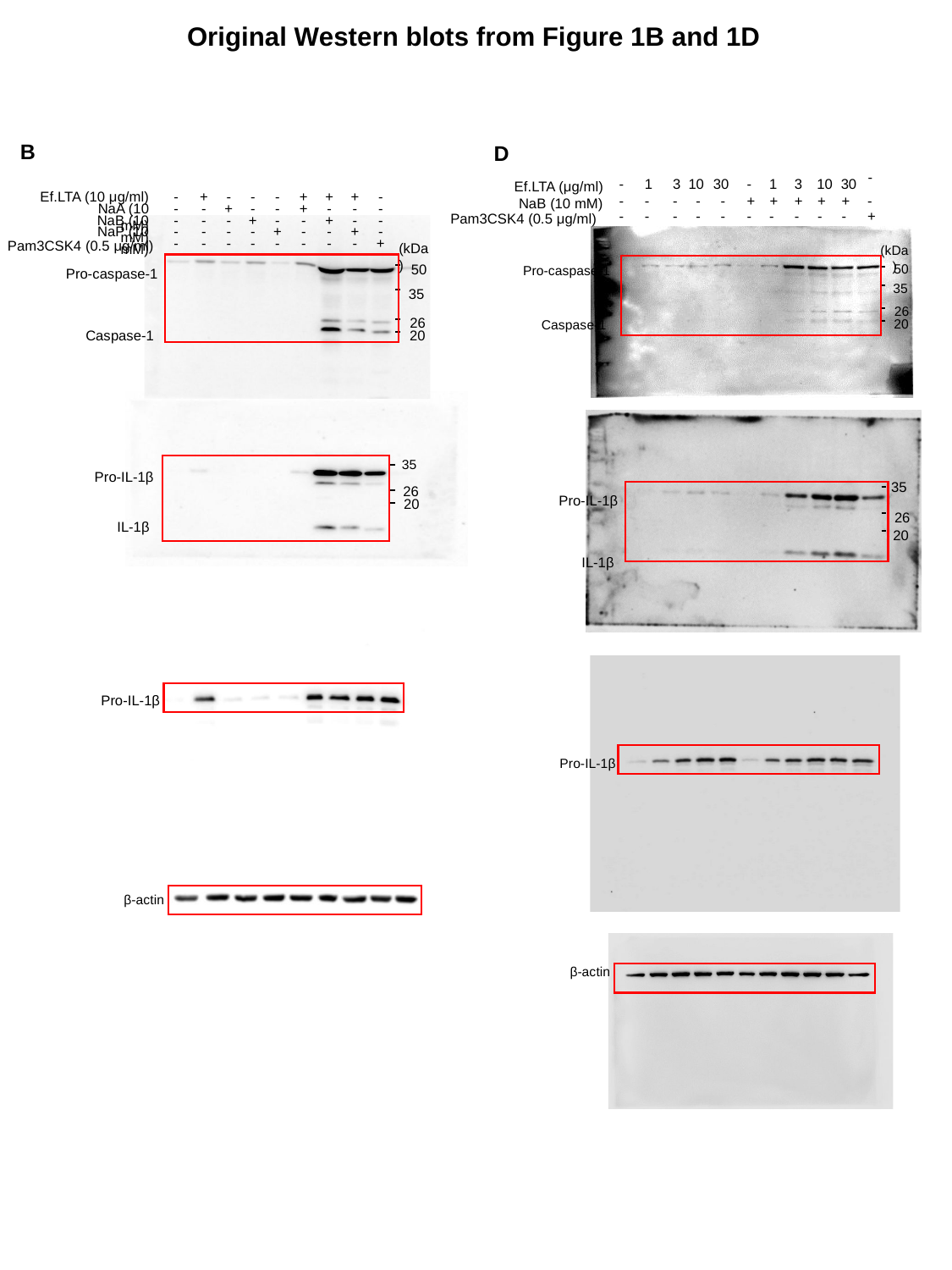

Original Western blots from Figure 1B and 1D
B
D
-
-
1
3
10
30
-
1
3
10
30
Ef.LTA (μg/ml)
Ef.LTA (10 μg/ml)
-
+
-
-
-
+
+
+
-
-
-
-
-
-
+
+
+
+
-
+
NaB (10 mM)
NaA (10 mM)
-
-
+
-
-
+
-
-
-
+
-
-
-
-
-
-
-
-
-
-
Pam3CSK4 (0.5 μg/ml)
NaB (10 mM)
-
-
-
+
-
-
+
-
-
(kDa)
50
Pro-caspase-1
35
26
20
Caspase-1
NaP (10 mM)
-
-
-
-
+
-
-
+
-
-
-
-
-
-
-
-
-
+
Pam3CSK4 (0.5 μg/ml)
(kDa)
+
-
-
-
-
-
-
-
-
-
-
50
Pro-caspase-1
35
26
20
Caspase-1
Pro-IL-1β
26
20
IL-1β
35
35
Pro-IL-1β
26
20
IL-1β
Pro-IL-1β
Pro-IL-1β
β-actin
β-actin

## Slide 2
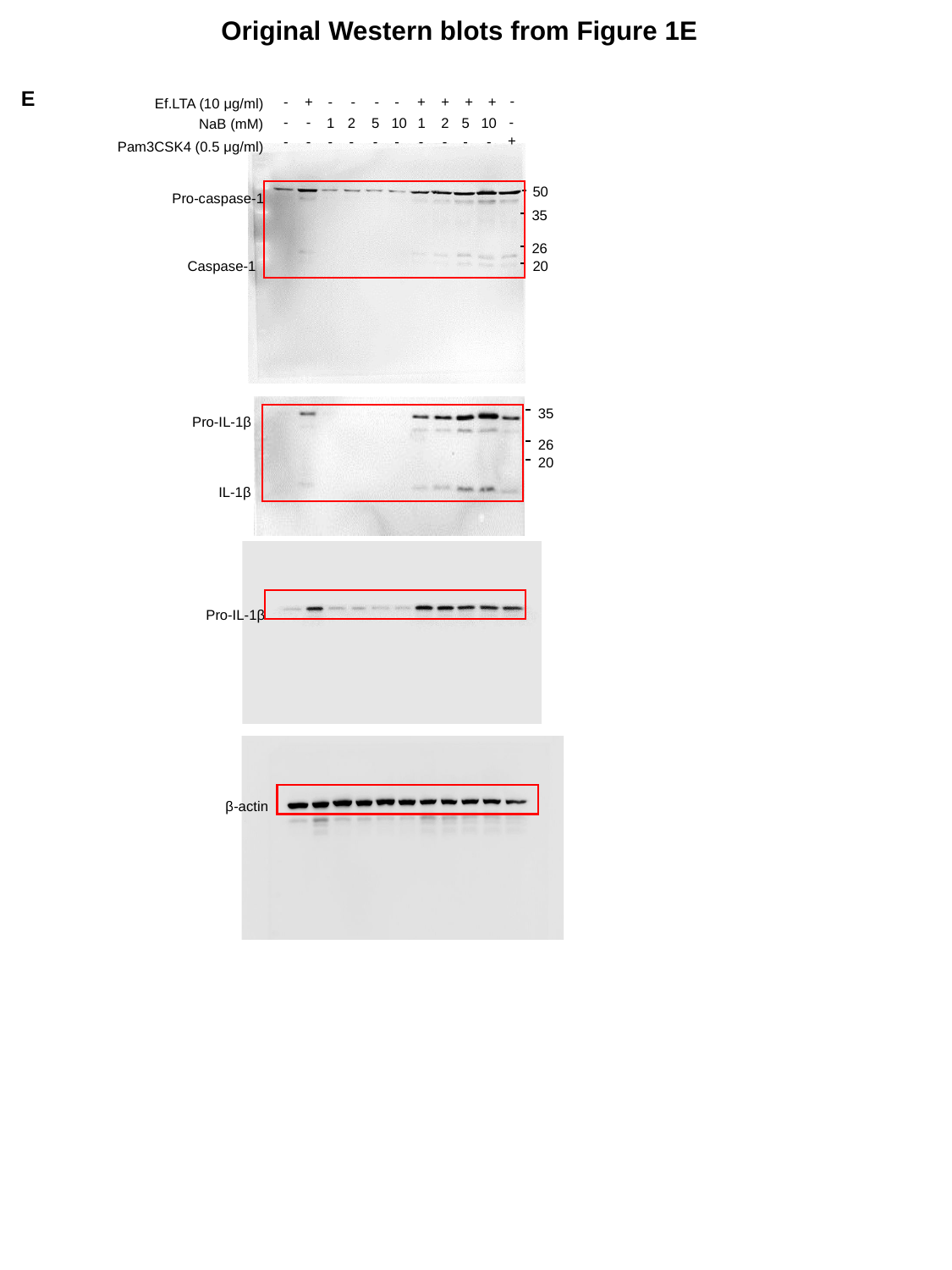

Original Western blots from Figure 1E
E
-
-
-
+
-
-
-
+
+
+
+
Ef.LTA (10 μg/ml)
-
-
-
10
10
1
2
5
1
2
5
NaB (mM)
+
-
-
-
-
-
-
-
-
-
-
Pam3CSK4 (0.5 μg/ml)
50
Pro-caspase-1
35
26
20
Caspase-1
35
Pro-IL-1β
26
20
IL-1β
Pro-IL-1β
β-actin

## Slide 3
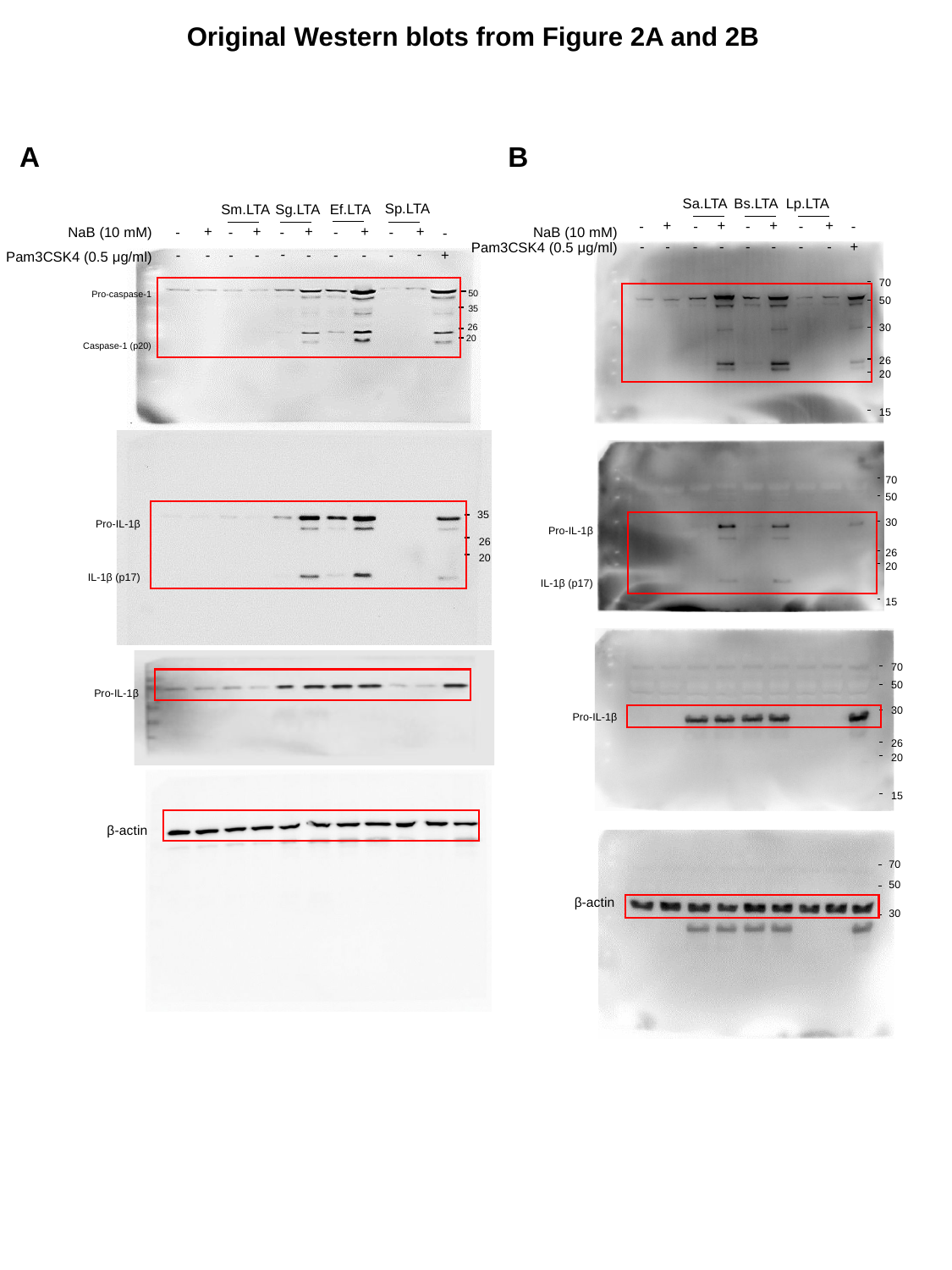

Original Western blots from Figure 2A and 2B
A
B
Sa.LTA
Bs.LTA
Lp.LTA
Sp.LTA
Sm.LTA
Ef.LTA
Sg.LTA
+
+
+
+
-
-
-
-
-
+
+
+
+
+
-
-
-
-
-
NaB (10 mM)
NaB (10 mM)
-
-
-
-
-
-
-
-
-
+
Pam3CSK4 (0.5 μg/ml)
-
-
-
-
-
-
-
-
-
-
+
Pam3CSK4 (0.5 μg/ml)
50
Pro-caspase-1
35
26
20
Caspase-1 (p20)
35
Pro-IL-1β
26
20
IL-1β (p17)
Pro-IL-1β
β-actin
70
50
30
26
20
15
70
50
30
Pro-IL-1β
26
20
IL-1β (p17)
15
70
50
30
Pro-IL-1β
26
20
15
70
50
β-actin
30

## Slide 4
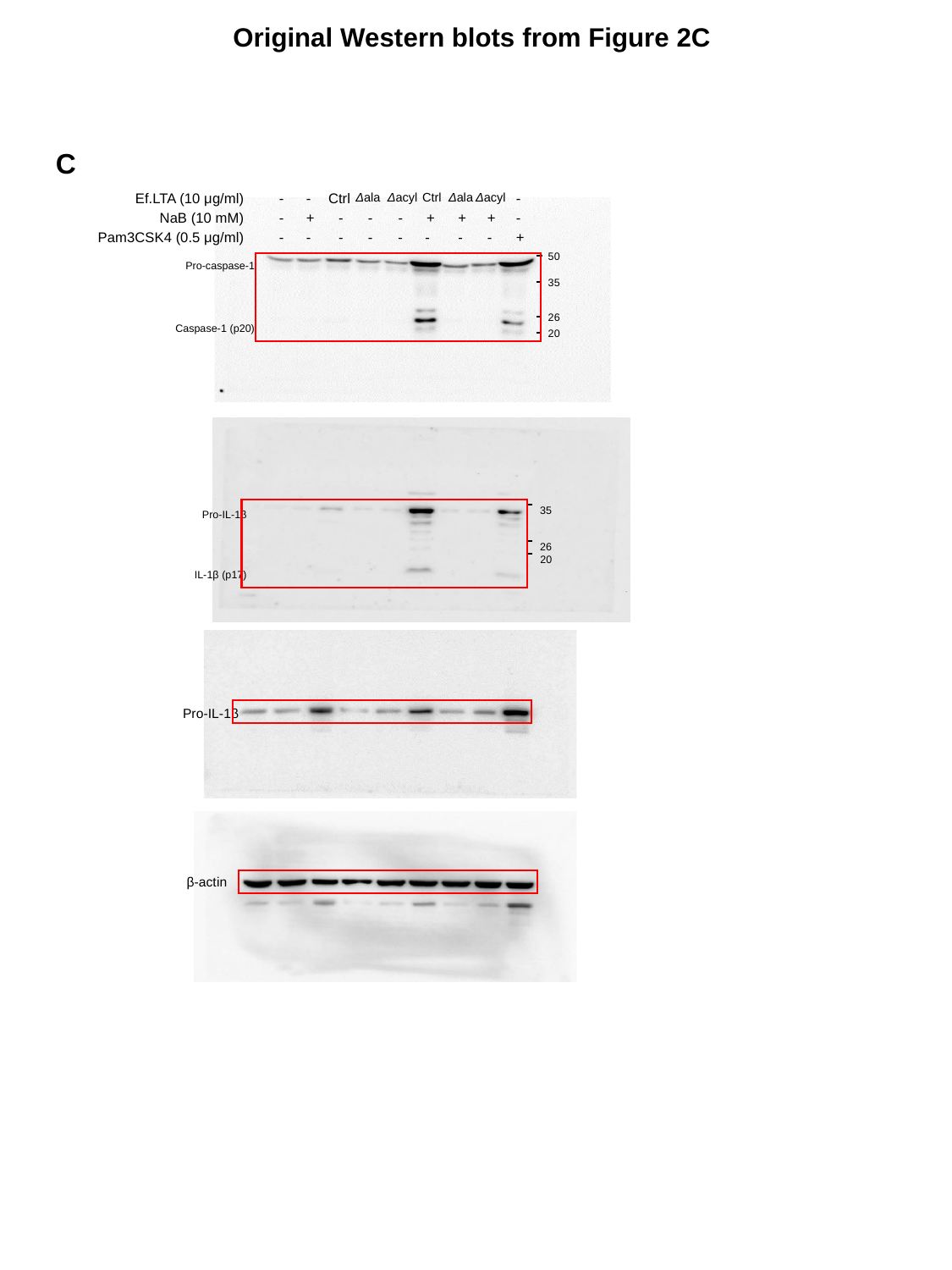

Original Western blots from Figure 2C
C
Ctrl
Δala
Δacyl
Ctrl
Δala
Δacyl
Ef.LTA (10 μg/ml)
-
-
-
50
Pro-caspase-1
35
26
Caspase-1 (p20)
20
35
Pro-IL-1β
26
20
IL-1β (p17)
Pro-IL-1β
β-actin
NaB (10 mM)
-
+
-
-
-
+
+
+
-
Pam3CSK4 (0.5 μg/ml)
-
-
-
-
-
-
-
-
+

## Slide 5
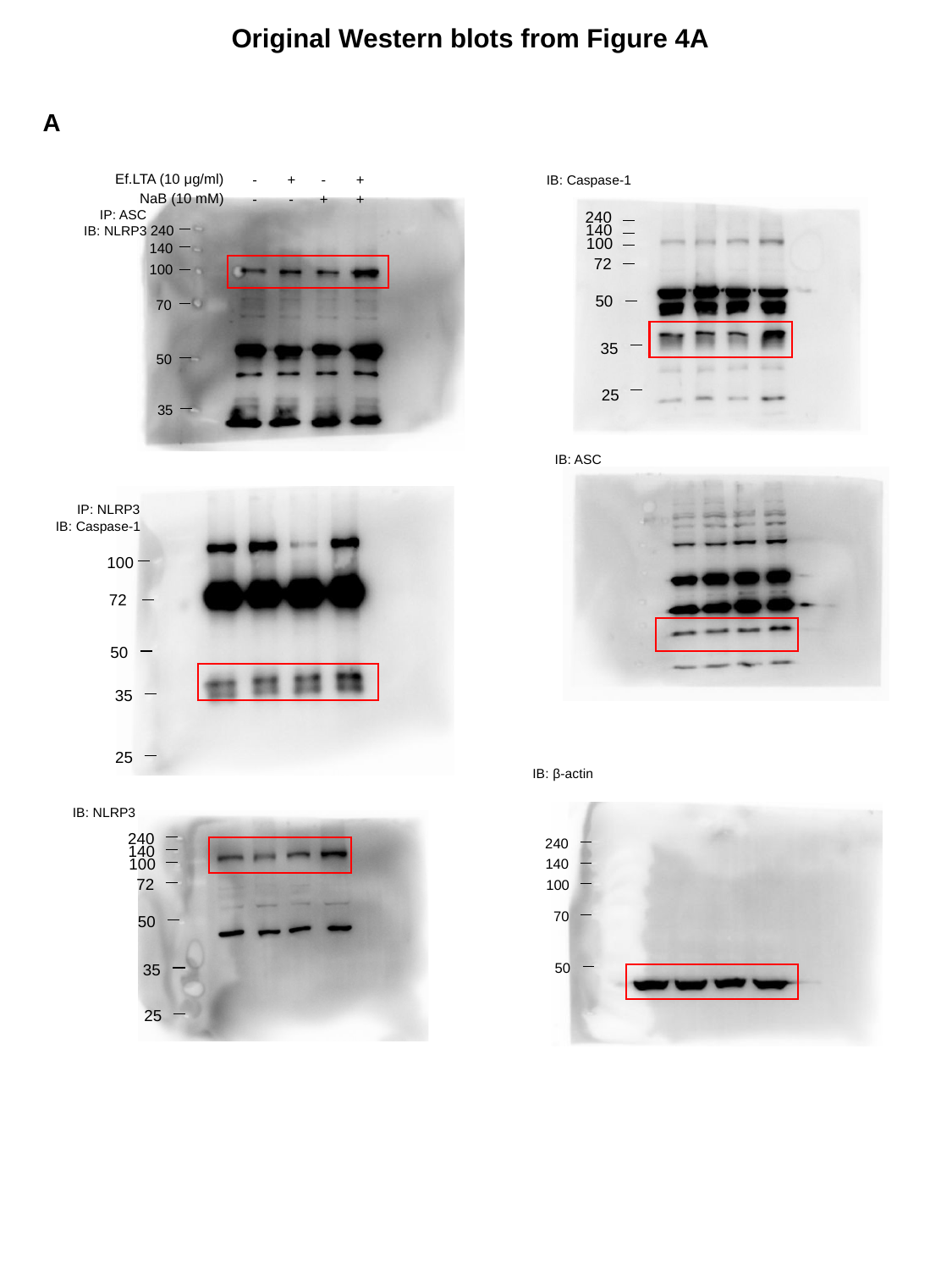

Original Western blots from Figure 4A
A
Ef.LTA (10 μg/ml)
-
+
-
+
IB: Caspase-1
NaB (10 mM)
-
-
+
+
240
140
100
70
50
35
IP: ASC
240
140
IB: NLRP3
100
72
50
35
25
IB: ASC
IP: NLRP3
IB: Caspase-1
100
72
50
35
25
IB: β-actin
IB: NLRP3
240
140
100
70
50
240
140
100
72
50
35
25

## Slide 6
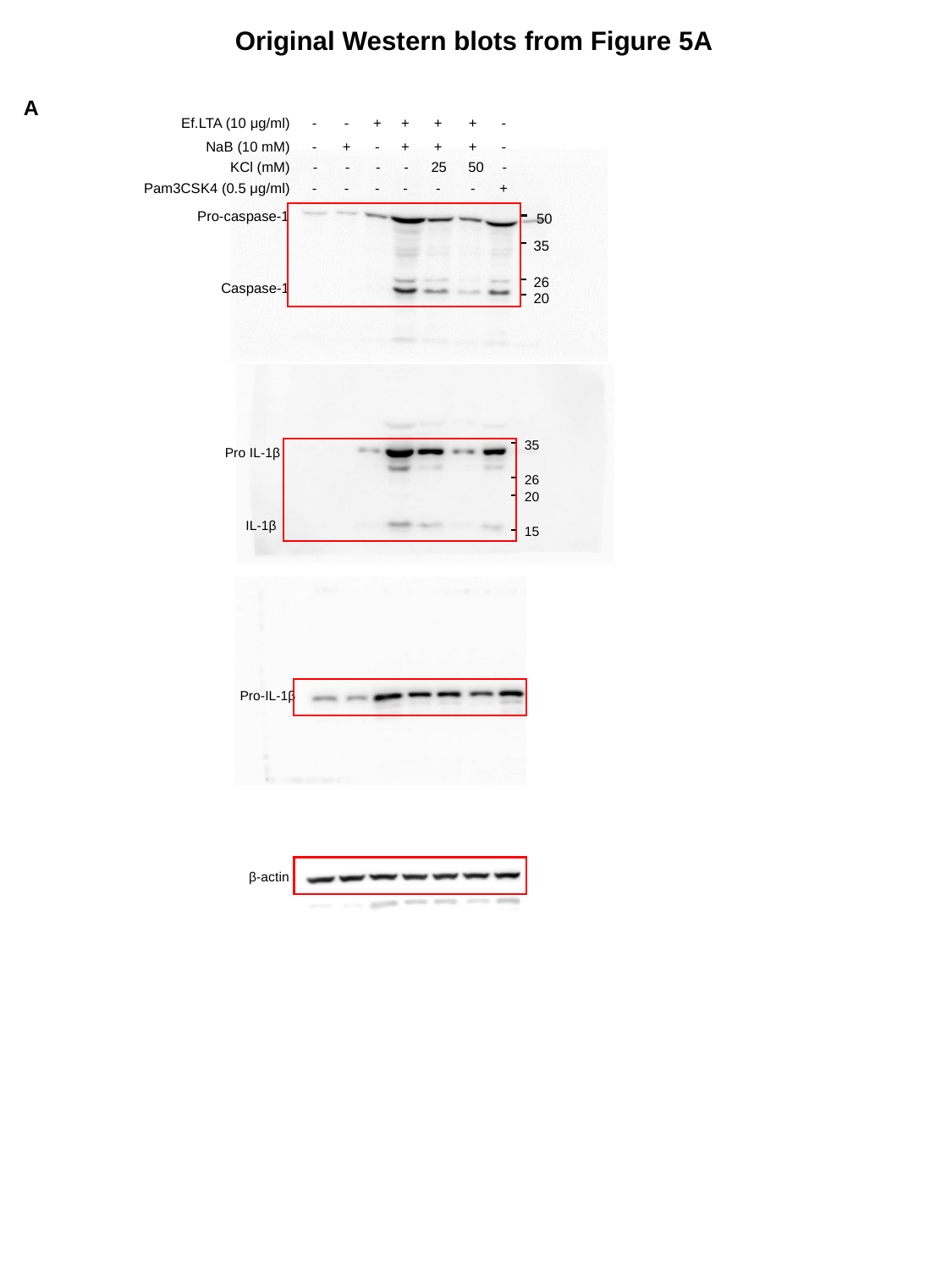

Original Western blots from Figure 5A
A
Ef.LTA (10 μg/ml)
-
-
+
+
+
+
-
NaB (10 mM)
-
+
-
+
+
+
-
Pro-caspase-1
50
35
26
Caspase-1
20
KCl (mM)
-
-
-
-
25
50
-
Pam3CSK4 (0.5 μg/ml)
-
-
-
-
-
-
+
35
Pro IL-1β
26
20
IL-1β
15
Pro-IL-1β
β-actin

## Slide 7
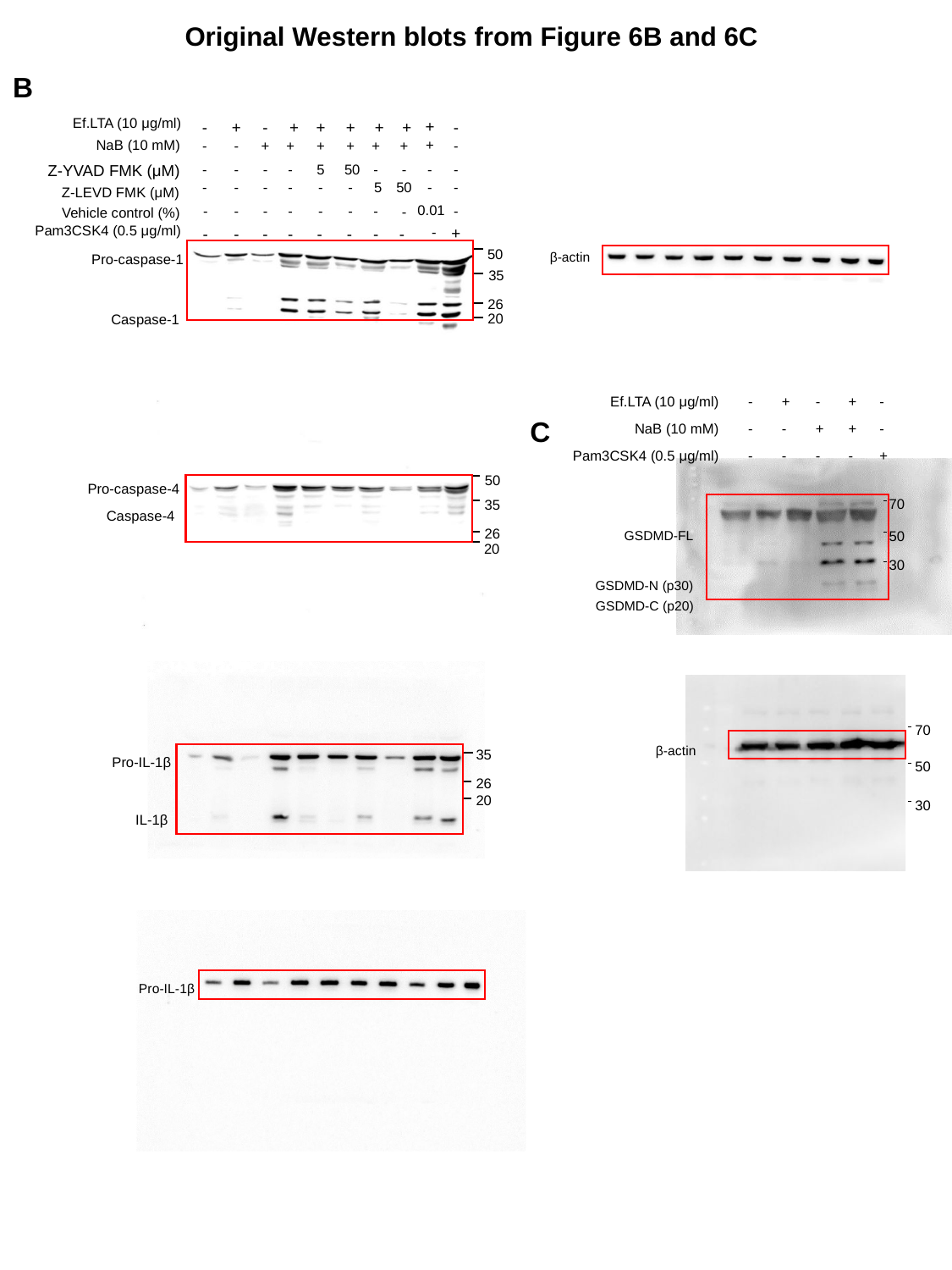

Original Western blots from Figure 6B and 6C
B
Ef.LTA (10 μg/ml)
+
-
-
+
-
+
+
+
+
+
NaB (10 mM)
+
-
-
-
+
+
+
+
+
+
-
-
-
-
-
5
-
-
50
Z-YVAD FMK (μM)
-
-
-
-
-
-
-
-
5
-
50
Z-LEVD FMK (μM)
0.01
-
-
-
-
-
-
-
-
-
Vehicle control (%)
Pam3CSK4 (0.5 μg/ml)
-
+
-
-
-
-
-
-
-
-
50
β-actin
Pro-caspase-1
35
26
20
Caspase-1
Ef.LTA (10 μg/ml)
-
+
-
+
-
C
NaB (10 mM)
-
-
+
+
-
50
Pro-caspase-4
35
Caspase-4
26
20
Pam3CSK4 (0.5 μg/ml)
-
-
-
-
+
70
GSDMD-FL
50
30
GSDMD-N (p30)
GSDMD-C (p20)
35
Pro-IL-1β
26
20
IL-1β
70
β-actin
50
30
Pro-IL-1β

## Slide 8
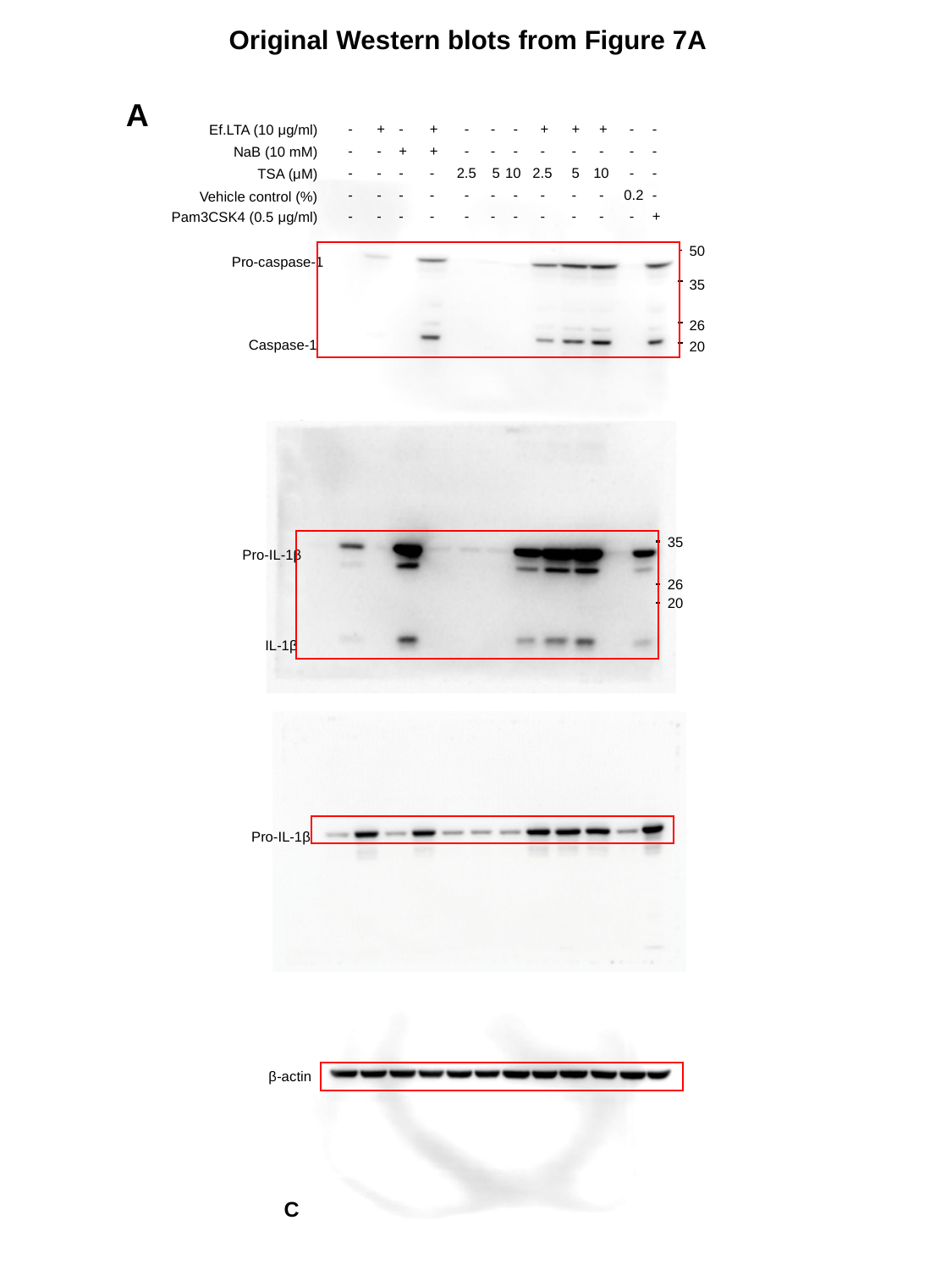

Original Western blots from Figure 7A
A
-
+
-
+
-
-
-
+
+
+
-
-
Ef.LTA (10 μg/ml)
-
-
+
+
-
-
-
-
-
-
-
-
NaB (10 mM)
5
10
5
-
-
-
-
-
-
2.5
10
2.5
TSA (μM)
50
Pro-caspase-1
35
26
Caspase-1
20
-
-
-
-
-
-
-
-
-
-
-
0.2
Vehicle control (%)
-
-
-
-
-
-
-
-
-
-
-
+
Pam3CSK4 (0.5 μg/ml)
-
35
Pro-IL-1β
26
20
IL-1β
Pro-IL-1β
β-actin
C

## Slide 9
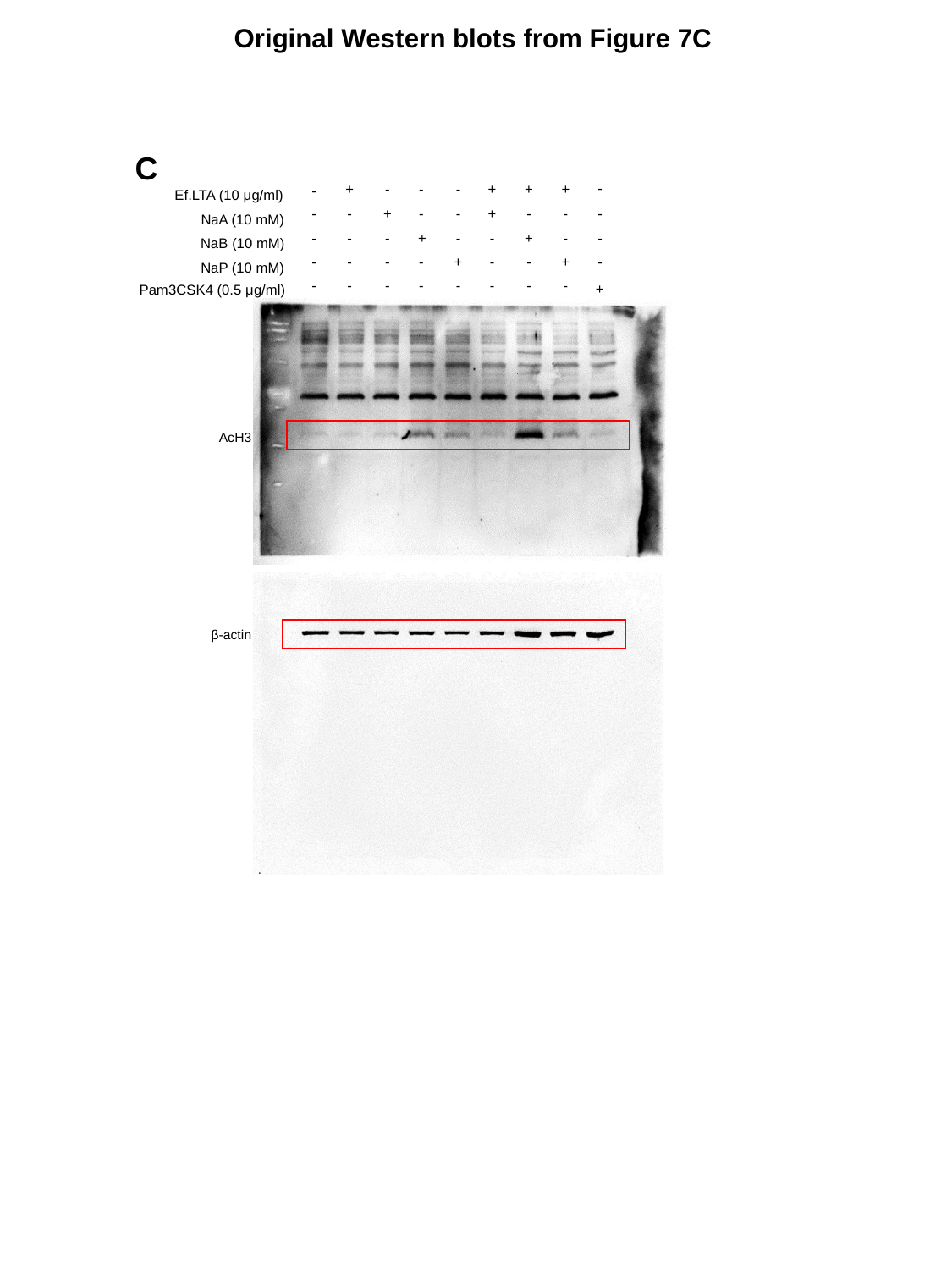

Original Western blots from Figure 7C
C
-
+
-
-
-
+
+
+
-
Ef.LTA (10 μg/ml)
-
-
+
-
-
+
-
-
-
NaA (10 mM)
-
-
-
+
-
-
+
-
-
NaB (10 mM)
-
-
-
-
+
-
-
+
-
NaP (10 mM)
-
-
-
-
-
-
-
-
+
Pam3CSK4 (0.5 μg/ml)
AcH3
β-actin
